# ARCaDia-selected short DNA core positions a latent warhead for protein-gated covalent capture

**DOI:** 10.1039/d6cb00303f

**Published:** 2026-10-16

**Authors:** Chaohui Li, Naoyuki Miyashita, Masayasu Kuwahara, Jay Yang, Masumi Taki

**Affiliations:** a Department of Engineering Science, The Graduate School of Informatics and Engineering, The University of Electro-Communications (UEC), 1-5-1 Chofugaoka, Chofu Tokyo 182-8585 Japan jyang75@wisc.edu taki@pc.uec.ac.jp +81-42-443-5980; b Department of Biological Systems Engineering, Graduate School of Biology-Oriented Science and Technology, KINDAI University, 930 Nishimitani, Kinokawa Wakayama 649-6493 Japan; c Graduate School of Integrated Basic Sciences, Nihon University, Setagaya-ku Tokyo 156-8550 Japan; d School of Medicine and Public Health, University of Wisconsin Madison Wisconsin 53706 USA; e Institute for Advanced Science, UEC Chofu 182-8585 Japan

## Abstract

A single-round affinity/reactivity co-selection of aptamer (ARCaDia), combined with Fast String-Based Clustering (FSBC) analysis of the selection output, identified a compact 10-nucleotide DNA-warhead core motif for streptavidin-selective covalent capture, showing that protein-gated covalent chemistry can arise from a short sequence-dependent placement of a latent warhead in a biotin-sensitive reaction microenvironment. This demonstrates that functional warhead-positioning information is already present after a single selection round.

## Abstract 2

ARCaDia-selected short DNA core positions a latent fosylate-type warhead for protein-gated covalent capture.

## Graphical abstract

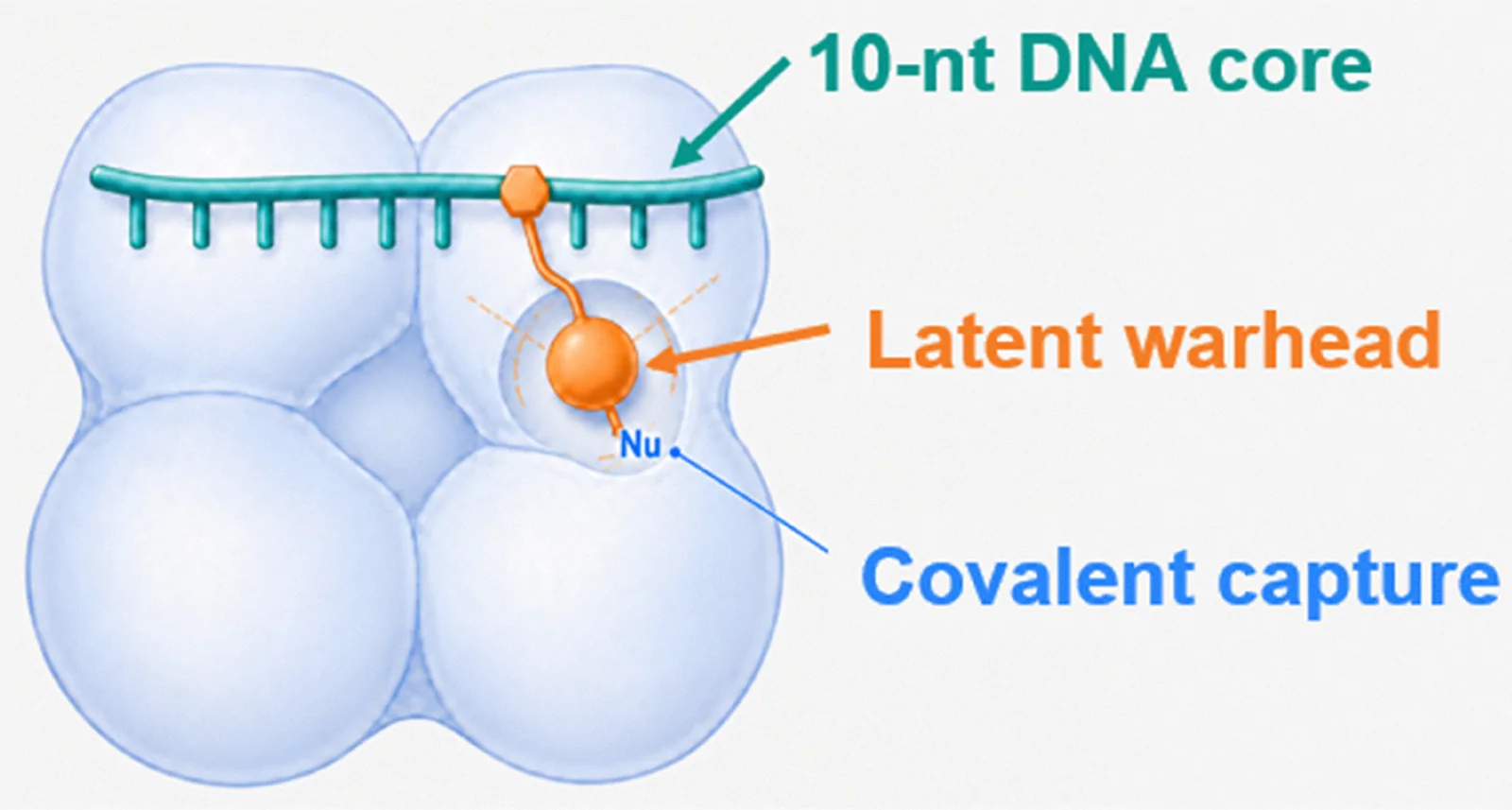

Targeted covalent inhibitors (TCIs) convert reversible molecular recognition into durable target engagement. Their central design problem is kinetic discrimination: bond formation should be accelerated at the target protein while reaction with solvent, serum proteins and off-target nucleophiles remains suppressed.^1,2^ This problem becomes especially important for middle-to-macro biomolecular TCIs (bioTCIs), where sequence-defined peptide, protein and nucleic-acid scaffolds can encode both target recognition and warhead presentation.^3,4^ Representative recent examples now span peptidic bioTCIs, including phage- and other display-selected covalent peptides,^5–7^ protein bioTCIs based on genetically encoded proximity-enhanced reactivity and fast-acting covalent protein platforms,^8–11^ and nucleotidic bioTCIs^12^ developed through covalent selection^13–19^ or post-selection covalent aptamer engineering.^20–26^

A common mechanistic requirement across these systems is that intrinsic warhead electrophilicity must be balanced against target-gated activation. Latent electrophiles, such as sulfur(vi) fluoride exchange (SuFEx) aryl fluorosulfates (AFS),^27^ are attractive in this respect because their effective reactivity depends strongly on proximity, geometry, and the local protein microenvironment rather than on bulk electrophilicity alone.^28–32^ Consistent with recent analyses of SuFEx-modified nucleic acids, productive covalent capture is not solely determined by affinity or warhead structure in isolation, but rather emerges from a matchmaking reaction microenvironment consisting of the coupled arrangements of the scaffold, target pocket, and latent electrophile.^33^ Among nucleotidic bioTCIs, recent SuFEx-based combinatorial screening (*i.e.*, covalent SELEX) studies^16,25^ are especially informative, because they use aptameric scaffolds not only as binding modules but also as sequence-encoded presenters/positioners of latent sulfur(vi) fluoride electrophiles. These studies have shown that aptameric scaffolds can support target-gated activation, and that binding affinity alone does not reliably predict covalent product formation. A complementary question, however, remains less explored: how much DNA scaffold sequence information is already present after a single reactivity-first selection round, before iterative enrichment or mature aptamer optimisation has occurred?

Building on our previous single-round affinity/reactivity co-selection (ARC) study, here we ask whether bioinformatic analysis of the selection output can identify a compact sequence-warhead arrangement sufficient to position a latent warhead within a target-gated protein reaction microenvironment. Our ARC framework was initially developed using a T7-phage peptide library modified with a latent AFS warhead, establishing a selection logic in which survival required both target engagement and activation of a weakly reactive warhead in the matchmaking microenvironment.^5^ We subsequently extended this ARC logic to aptamer screening and established a single-round/non-SELEX system, namely ARCaDia, using a DNA library functionalised with one latent AFS warhead at a single defined position.^17^ Using streptavidin (SA) as a structurally defined model target,^15^ we now show that bioinformatic analysis of the single-round ARCaDia output identifies a compact 10-nucleotide (nt) DNA scaffold sufficient to position the latent warhead for SA-gated covalent capture.

Fig. 1A shows a conceptual workflow of the present experiments designed to identify a compact DNA scaffold capable of target-gated covalent capture after a single round of ARCaDia screening. We first performed ARCaDia against SA using the defined DNA library (Fig. S3), as previously described,^17^ and then analysed the HTS readouts by Fast String-Based Clustering (FSBC)^34^ rather than relying on conventional fixed-length *k*-mer^17,35^ or full-length readout abundance counting (Fig. 1B). Simple *k*-mer analysis reports what is frequent (Table S2, SI), whereas FSBC asks what local sequence information is recurrent enough to define an experimentally testable candidate local sequence cluster/motif. This distinction was critical here: single-round ARCaDia was designed not to recover a mature full-length aptamer family, but to reveal a compact warhead-proximal DNA scaffold capable of positioning latent chemistry within a target-gated reaction microenvironment. FSBC identified 8–10-nt strings, and alignment of the full 25-nt readouts containing the highest-scoring short motif GCTTCGATGC revealed a conserved short core flanked by variable 5′ and 3′ regions (Fig. 1C). Because the AFS-modified U* was placed at the 13th nucleotide between two N_12_ random regions in the starting library, and this fixed position mapped predominantly to the 8th nucleotide of the aligned seed-containing reads (Table S3, SI), this motif was assigned as GCTTCGAU*GC and tested directly as Core-AFS. Core-AFS retained SA-dependent covalent capture in the presence of human serum, whereas reverse-order controls with the warhead in the 3rd or 8th position (Rev_3_ or Rev_8_, respectively), with matched length, nucleotide composition, and the same AFS-modified nucleotide, did not (Fig. 2A). Notably, Rev_8_-AFS retained U* at the same position as Core-AFS yet showed negligible covalent capture, indicating that warhead position alone is insufficient and supporting a critical role for the local sequence arrangement in productive reactivity. This covalent capture by Core was SA-specific: the closely related NeutrAvidin (Neut) and the unrelated lysozyme (Lyso) showed negligible mobility shift on reducing SDS-PAGE (Fig. 2B). The Core-AFS reaction with SA was concentration-dependent and was well described by a Hill equation (Fig. 2C). Productive capture therefore cannot be explained simply by oligonucleotide charge alone,^36^ electrophile exposure, or the presence of AFS. Rather, it requires sequence-dependent placement of the latent AFS warhead within a matchmaking microenvironment by this short DNA scaffold to enable covalent reaction with the SA protein. Consistent with previous observations,^22^ Core-AFS covalently bound to SA demonstrated relative resistance to nuclease digestion (Fig. S4, SI).

**Fig. 1:**
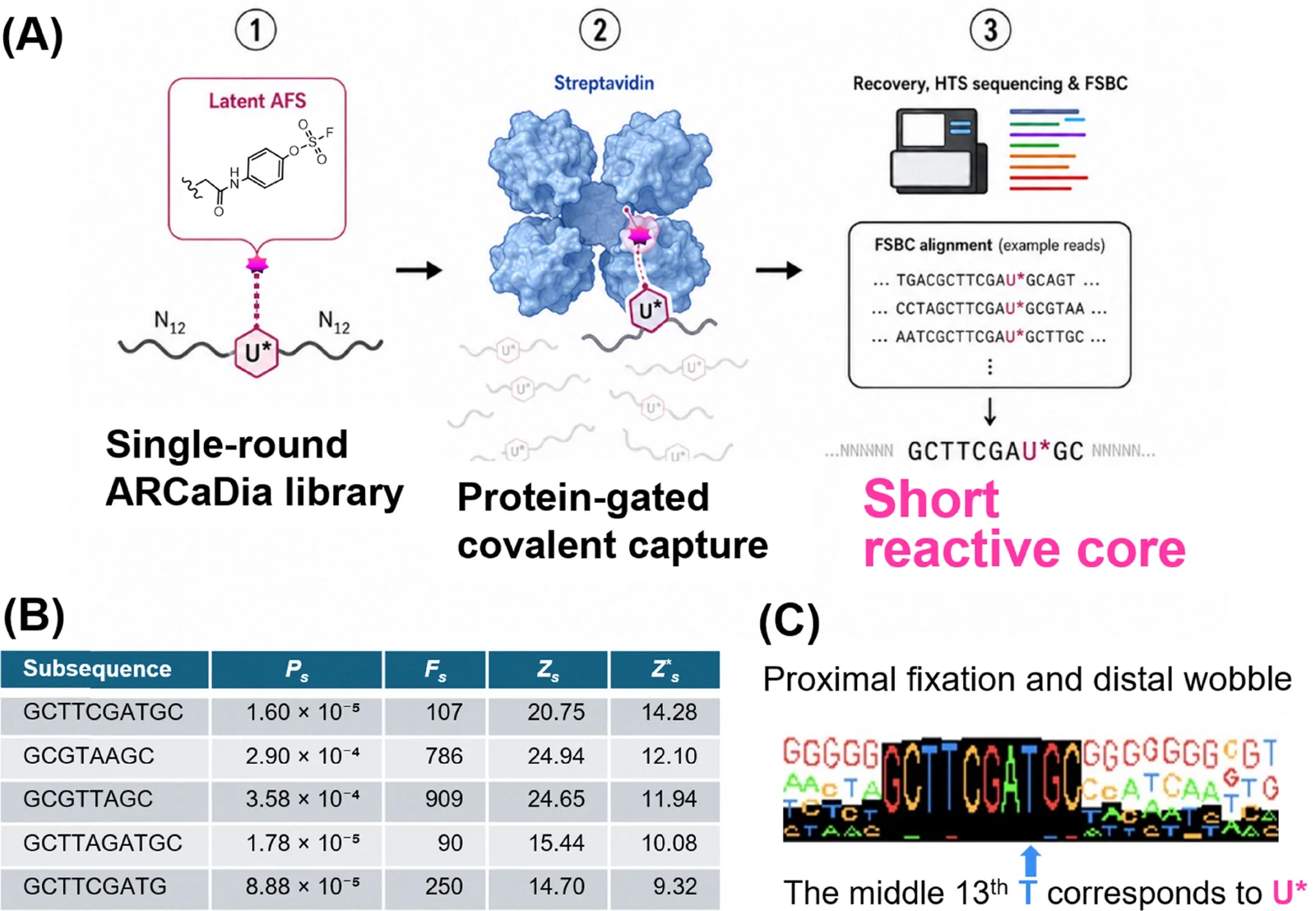
Conceptual workflow of single-round ARCaDia followed by FSBC bioinformatics analysis of HTS reads. (A) A latent-warhead DNA library consisting of a central octadiynyl residue modified with an AFS warhead (U*) flanked by N_12_ random sequences on both sides was subjected to ARCaDia against streptavidin, and the recovered pool was analysed by FSBC to identify a warhead-proximal compact reactive core. The AFS warhead is shown in the dotted square. (B) Post-selection FSBC output of top-ranked over-represented subsequences (*i.e.*, strings). For each subsequence s, *P*_s_, *F*_s_, *Z*_s_, and 
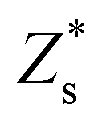
 denote the expected occurrence probability, observed read count, enrichment *Z*-score, and length-normalised ranking score, respectively. (C) Alignment of the 25-nucleotide (nt) read sequences containing the highest-scoring 10-nt core motif GCTTCGAU*GC (Core-AFS), characterised by proximal fixation and distal wobble. The U* position was assigned from the defined library design.

**Fig. 2:**
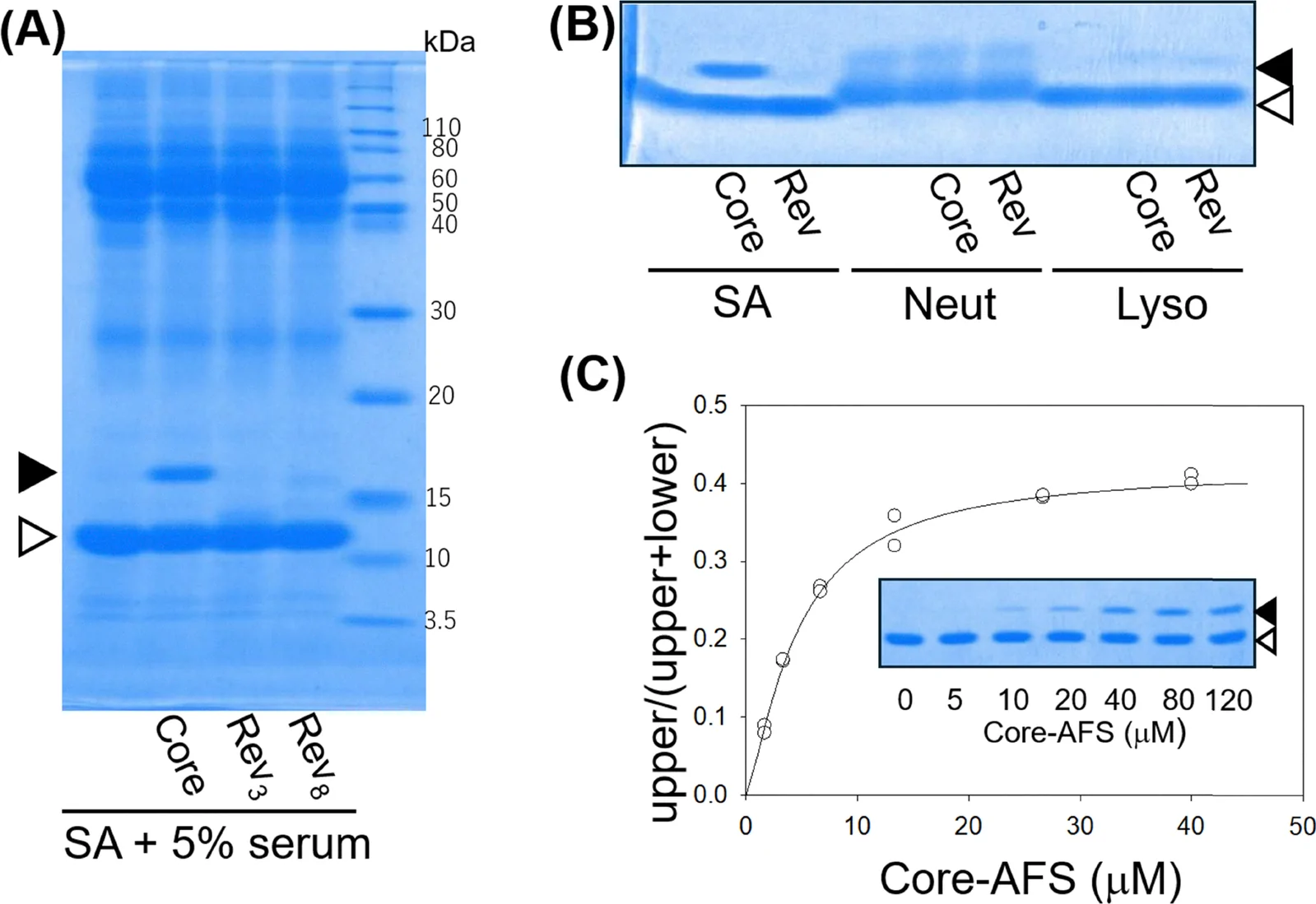
AFS-conjugated Core selectively forms a covalent adduct with SA. (A) Core-AFS, but not the reverse-order sequence (Rev_3_- or Rev_8_-AFS; Table S1, SI), produced a clear mobility-shifted SA covalent adduct (closed arrow) in the presence of 5% human serum. The non-shifted unreacted SA was seen as a monomer (open arrow) in this reducing SDS-PAGE. All subsequent experiments used Rev_3_ as the negative control. (B) Same as A, except that Core-AFS or Rev-AFS was reacted with NeutrAvidin (Neut) or lysozyme (Lyso). (C) Concentration-dependent covalent capture of SA by Core-AFS, shown by SDS-PAGE (inset) and gel quantitation of the fraction, upper/(upper + lower), plotted against Core-AFS concentration. The solid line is a Hill equation fit to the data (open circles), fraction shift = *A*_max_[*C*]^*n*^/(EC_50_^*n*^ + [*C*]^*n*^), with *A*_max_ = 0.42, *n* = 1.3, EC_50_ = 4.57 ± 0.27 µM, all *P* < 0.001. Experiments in (A) and (B) were performed with SA or other target proteins at 20 µM and Core-AFS or Rev-AFS at 90 µM for 24 h at 37 °C; in (C), Core-AFS was varied as indicated.

Time-dependent formation of the mobility-shifted SA band was quantified by reducing SDS-PAGE. For each Core-AFS concentration, the shifted-band fraction increased with time and was empirically fitted to a mono-exponential approach to plateau, yielding an apparent first-order rate constant, *k*_obs_. At 10 µM Core-AFS, *k*_obs_ was 0.15 h^−1^ (Fig. 3A). To interpret the concentration dependence of *k*_obs_, we first considered the classical two-step irreversible-binding scheme shown in the dashed box in Fig. 3B, in which reversible complex formation precedes covalent conversion.^37^ However, the data did not follow the simple hyperbolic saturation expected for this model (Fig. 3B and C). Instead, *k*_obs_ reached a maximum at low micromolar Core-AFS concentrations and decreased thereafter, indicating that increasing Core-AFS does not simply increase the population of productive reaction complexes. This non-hyperbolic behaviour is consistent with the existence of one or more concentration-dependent less-reactive states, such as oligonucleotide self-association, secondary binding, or occupancy-dependent effects within tetrameric SA, which would reduce the apparent fraction of SuFEx-competent complexes at elevated Core-AFS concentrations. A phenomenological partial-substrate-inhibition model^38,39^ reproduced the overall trend (Fig. 3C, red line), but multiple physical mechanisms (Fig. S5, SI) can account for the observed suppression. Because these alternatives cannot be distinguished by the present experiments, the fitted parameters should be regarded as operational kinetic descriptors rather than true microscopic kinetic parameters and do not confirm that partial-substrate inhibition is the underlying mechanism. Nevertheless, the kinetic data support the conclusion that the single-round ARCaDia-derived 10-nt Core is sufficient for sequence-dependent, target-gated covalent conversion. At the same time, the slow apparent conversion and non-hyperbolic concentration dependence indicate that this Core is not yet an optimised high-affinity covalent aptamer, but rather a compact warhead-positioning scaffold.

**Fig. 3:**
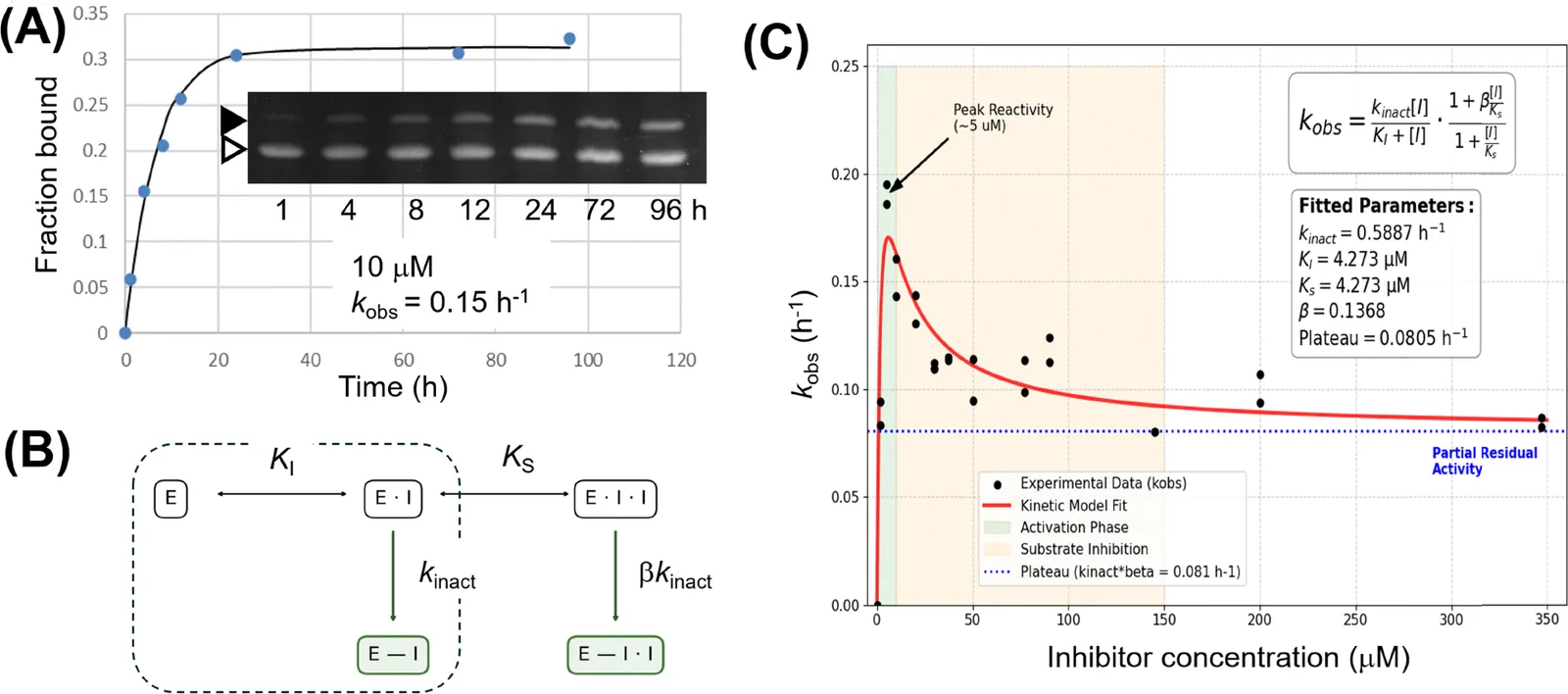
Kinetic analysis of the Core-AFS covalent reaction with SA. (A) SDS-PAGE (Oriole staining) of Core-AFS (10 µM) reacted with SA (6.7 µM) for the indicated times (h), with gel quantification used to obtain the fraction bound (upper/(upper + lower)) fitted to a mono-exponential equation *f*(*t*) = *f*_∞_(1−e^−*k*_obs_·*t*^). (B) Kinetic model used to describe the concentration dependence of *k*_obs_. The scheme shows the kinetic model in which *I* denotes Core-AFS and *E* denotes SA; the boxed three-state model corresponds to the canonical reversible complex formation followed by an irreversible covalent conversion. Additional states representing partial substrate inhibition were included to reproduce the concentration-dependent suppression of *k*_obs_. (C) Apparent *k*_obs_ obtained at various Core-AFS concentrations. Each dot indicates an independent experiment, with at least duplicate measurements for each concentration. The red curve shows the best fit to the phenomenological partial-substrate-inhibition model. Fitting used a weighting factor *W* = (*k*_obs_ + 0.05)^2^.

The biotin-binding architecture of tetrameric SA therefore provided a means to test whether Core-AFS capture was associated with the biotin-binding pockets. SA exists as a homo-tetramer and binds biotin at well-defined pockets deep within the β-barrel, with contributions from residues at the subunit interface.^40,41^ A biotin competition experiment demonstrated that biotin suppressed Core-AFS capture, but only when applied before Core-AFS (Fig. 4A and Fig. S6, SI). Although these experiments do not provide residue-level assignments of the conjugated amino acids, they argue against non-specific protein labelling and support warhead placement near a biotin-sensitive region of SA.^42,43^

**Fig. 4:**
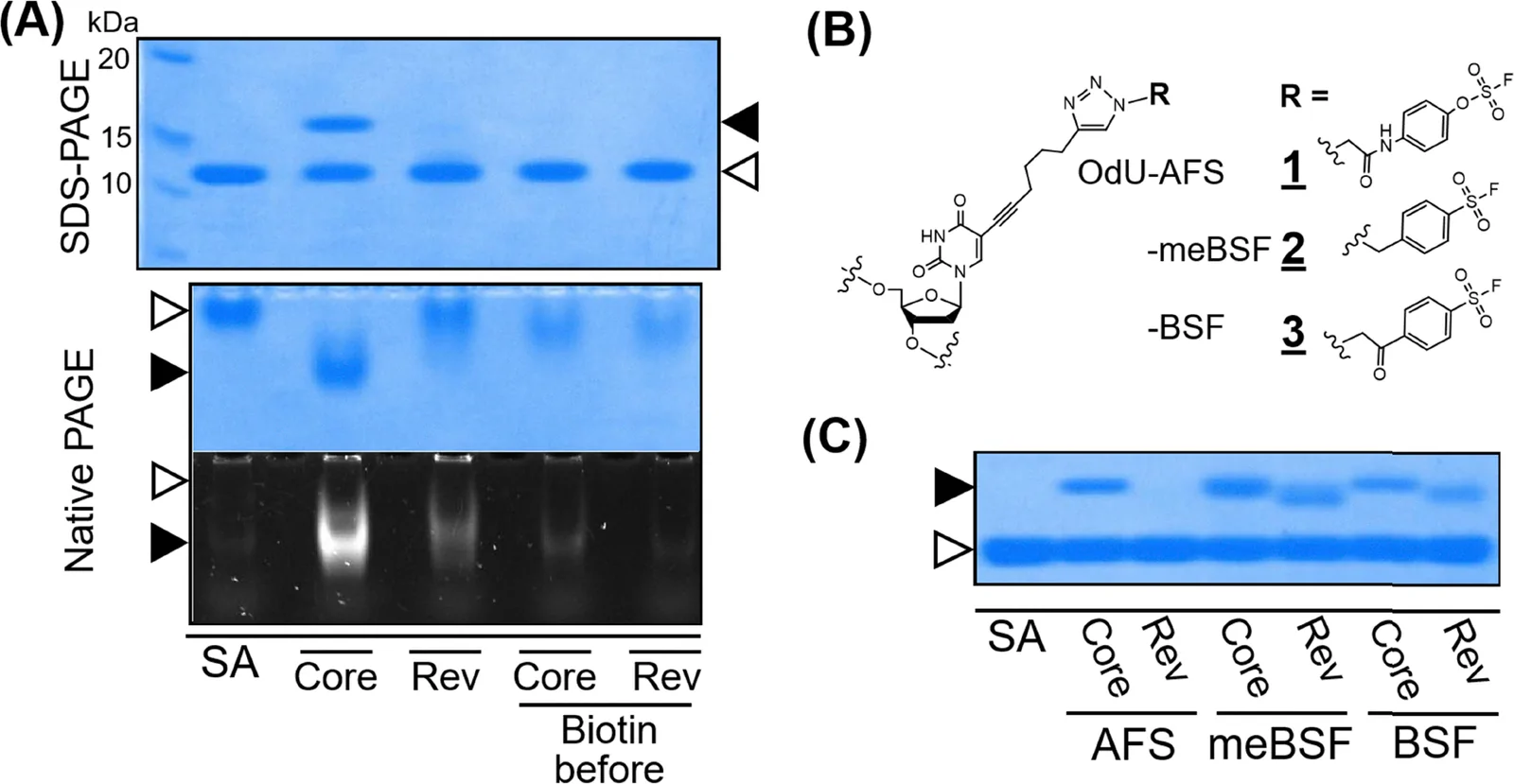
The selected 10-nt Core positions the latent AFS warhead for biotin-sensitive, SA-gated covalent capture. (A) Core-AFS or Rev-AFS was reacted with SA in the absence of biotin, except for the lanes indicated as “Biotin before”, in which SA was pre-incubated with a two-fold molar excess of biotin relative to SA monomer before addition of the oligonucleotide conjugate. Each reaction mixture was analysed by both denaturing SDS-PAGE and native PAGE. On SDS-PAGE, the lower band is the unreacted SA (open arrow) and the mobility-shifted upper band is the covalently modified SA monomer (closed arrow). On native PAGE, unmodified tetrameric SA is indicated by an open arrow and mobility-shifted tetrameric SA by a closed arrow; the two panels are the same gel stained with CBB for protein (upper) and SYBR Gold for DNA (lower). (B) Structures of the octadiynyl (OdU)-linked warheads installed by click conjugation: AFS (**1̲**), meBSF (**2̲**), and BSF (**3̲**). (C) Core and Rev modified with AFS, meBSF, or BSF were reacted with SA in the same manner and analysed by SDS-PAGE. AFS gave the clearest Core-over-Rev discrimination, whereas meBSF and BSF increased Rev-mediated background capture.

The Core-AFS-induced, biotin-sensitive mobility-shifted SA band on SDS-PAGE accounted for approximately half of the total SA signal (Fig. 2B and 4A, lane SA/Core), and we asked whether this reflected incomplete covalent reaction at the tetramer level or a monomer-level readout of tetrameric SA. We therefore analysed the same reaction mixtures in parallel by denaturing SDS-PAGE and native PAGE (Fig. 4A). In denaturing SDS-PAGE, Core-AFS reproducibly gave shifted and unshifted SA subunit bands at an approximately 1 : 1 ratio (Fig. 4A, top). In contrast, native PAGE, where the oligomeric SA is preserved, showed that Core-AFS induced a complete mobility shift of oligomeric SA, whereas Rev-AFS did not, and this shift was suppressed by biotin preincubation (Fig. 4A, middle). Thus, the 50% mobility-shifted fraction on SDS-PAGE does not reflect an incomplete tetramer-level capture but is consistent with the dissociation of Core-AFS-modified tetramers into conjugated and unconjugated monomers. Furthermore, the observed mobility shift of the monomer is consistent with a single Core-AFS modification per shifted SA monomer. Together, these data are consistent with an average of approximately two Core-AFS modifications per SA tetramer.

To further examine whether Core-dependent covalent capture required a matched warhead-latency window, rather than simply increasing with intrinsic electrophilicity,^5,28^ we compared three sulfur(vi) fluoride variants^29–32^ on the same Core and Rev scaffolds (Fig. 4B): the latent AFS,^5,17^ a less latent methylene-linked benzenesulfonyl fluoride (meBSF),^23^ and the most intrinsically reactive carbonyl-linked benzenesulfonyl fluoride (BSF).^20,22^ AFS gave the clearest sequence discrimination, whereas both meBSF and BSF increased background capture with Rev, consistent with partial collapse of sequence-dependent gating under the tested conditions (Fig. 4C). Thus, within the Core–Rev scaffold pair, AFS provided the best balance between productive SA capture and suppression of sequence-independent background reactivity.

In summary, FSBC analysis of single-round ARCaDia output identified a compact 10-nt warhead-proximal DNA scaffold capable of SA-gated covalent capture. Sequence reversal, biotin competition, serum-containing reactions, non-target controls with lysozyme and NeutrAvidin, and warhead comparison together show that productive capture arises from sequence-dependent warhead placement/orientation in a biotin-sensitive reaction microenvironment, rather than from non-specific electrophile exposure. Thus, the single-round ARCaDia coupled with FSBC enabled extraction of local sequence-warhead information defining a compact scaffold that supports target-gated reactivity without constituting a mature high-affinity aptamer. If similar compact sequences against biologically important targets can be found, analyses of the interactions between such tractable compact scaffolds and their respective targets may provide a mechanistic understanding at the molecular level of the key determinants governing target-gated reactivity. In turn, this could enhance our ability to design high-affinity covalent aptameric drugs.

## Author contributions

C. L., J. Y., and M. T.: conceptualization, investigation, and writing of the manuscript; N. M. and M. K.: review and editing. M. T.: funding acquisition.

## Conflicts of interest

There are no conflicts to declare.

## Supplementary Material

CB-OLF-D6CB00303F-s001

CB-OLF-D6CB00303F-s002

CB-OLF-D6CB00303F-s003

## Data Availability

All data supporting the findings of this study are included in the article and supplementary information (SI). The SI includes the raw HTS data as FASTQ files, the locally used pFSBC_custom.py script, and the details of the web-server-based analysis procedures. Several references are cited only in the SI.^44–46^ See DOI: https://doi.org/10.1039/d6cb00303f.

## References

[cit1] Singh J., Petter R. C., Baillie T. A., Whitty A. (2011). Nat. Rev. Drug Discovery.

[cit2] Boike L., Henning N. J., Nomura D. K. (2022). Nat. Rev. Drug Discovery.

[cit3] Yang J., Tabuchi Y., Katsuki R., Taki M. (2023). Int. J. Mol. Sci..

[cit4] Tamura T., Kawano M., Hamachi I. (2025). Chem. Rev..

[cit5] Tabuchi Y., Watanabe T., Katsuki R., Ito Y., Taki M. (2021). Chem. Commun..

[cit6] Wang S., Faucher F. F., Bertolini M., Kim H., Yu B., Cao L., Roeltgen K., Lovell S., Shanker V., Boyd S. D., Wang L., Bartenschlager R., Bogyo M. (2025). J. Am. Chem. Soc..

[cit7] Walker J. H., Yan K., Derda R. (2026). Biochemistry.

[cit8] Yu B., Li S., Tabata T., Wang N., Cao L., Kumar G. R., Sun W., Liu J., Ott M., Wang L. (2022). Chemistry.

[cit9] Xiang Z., Ren H., Hu Y. S., Coin I., Wei J., Cang H., Wang L. (2013). Nat. Methods.

[cit10] Li Q., Chen Q., Klauser P. C., Li M., Zheng F., Wang N., Li X., Zhang Q., Fu X., Wang Q., Xu Y., Wang L. (2020). Cell.

[cit11] Fan Q., Mei J., Li T., Zang C., Li M., Tang J., Xu Y., Yu G., Liu D., Chen K., Yang B., Huang J., Zhou T., Dang B. (2026). Science.

[cit12] Albright S., Boette J., Cacace M., Deiters A. (2026). RSC Chem. Biol..

[cit13] Smith D., Kirschenheuter G. P., Charlton J., Guidot D. M., Repine J. E. (1995). Chem. Biol..

[cit14] Charlton J., Kirschenheuter G. P., Smith D. (1997). Biochemistry.

[cit15] MacPherson I. S., Liedl T. (2018). Aptamers.

[cit16] Qin Z., Zhang K., He P., Zhang X., Xie M., Fu Y., Gu C., Zhu Y., Tong A., Wei H., Zhang C., Xiang Y. (2023). Nat. Chem..

[cit17] Taki M., Kuwahara M., Li C., Tomoda N., Miyashita N., Kan T., Yang J. (2024). Chem. Commun..

[cit18] Soxpollard N., Strauss S., Jungmann R., MacPherson I. S. (2024). Commun. Chem..

[cit19] Bian S., Qiu Q., Zhang C., Cao H., Xuan H., Yan X., Li J., Chen W., Liu X., Chen Y., Long J., Su H., Wang J., Zhang L. (2025). J. Am. Chem. Soc..

[cit20] Tabuchi Y., Yang J., Taki M. (2021). Chem. Commun..

[cit21] Tivon Y., Falcone G., Deiters A. (2021). Angew. Chem., Int. Ed..

[cit22] Tabuchi Y., Yang J., Taki M. (2022). Int. J. Mol. Sci..

[cit23] Wang D., Zhang J., Huang Z., Yang Y., Fu T., Yang Y., Lyu Y., Jiang J., Qiu L., Cao Z., Zhang X., You Q., Lin Y., Zhao Z., Tan W. (2023). ACS Cent. Sci..

[cit24] Jo H., Ju S., Kim M., Beon J., Jang S.-Y., Pack S. P., Son C. Y., Kim J.-S., Oh S. S. (2025). J. Am. Chem. Soc..

[cit25] Zhang K., Li J., Shi Y., Hou S., Qin Z., Shi W., Zhu Y., Zhang J., Tong A., Xiang Y. (2025). J. Am. Chem. Soc..

[cit26] Zhang Z., Qian H., Xu R., Duan Q., Wang Y., Fu T., Wu X., Sha C., Wu Q., Wang Y., Tan W. (2025). J. Am. Chem. Soc..

[cit27] Dong J., Krasnova L., Finn M. G., Sharpless K. B. (2014). Angew. Chem., Int. Ed..

[cit28] Frankovich T. V., McCann H. M., Hoffman K. S., Rullo A. F. (2025). ACS Cent. Sci..

[cit29] Jones L. H. (2025). Chem. Sci..

[cit30] Jones L. H. (2018). ACS Med. Chem. Lett..

[cit31] Zheng Q., Woehl J. L., Kitamura S., Santos-Martins D., Smedley C. J., Li G., Forli S., Moses J. E., Wolan D. W., Sharpless K. B. (2019). Proc. Natl. Acad. Sci. U. S. A..

[cit32] Hillebrand L., Liang X. J., Serafim R. A. M., Gehringer M. (2024). J. Med. Chem..

[cit33] Chromiński M., Żmudziński M., Jemielity J. (2026). RSC Chem. Biol..

[cit34] Kato S., Ono T., Minagawa H., Horii K., Shiratori I., Waga I., Ito K., Aoki T. (2020). BMC Bioinf..

[cit35] Hoon S., Zhou B., Janda K. D., Brenner S., Scolnick J. (2011). Biotechniques.

[cit36] Marson G., Palumbo M., Sissi C. (2012). Curr. Pharm. Des..

[cit37] Kitz R., Wilson I. B. (1962). J. Biol. Chem..

[cit38] Yoshino M., Murakami K. (2015). SpringerPlus.

[cit39] Lin Y., Lu P., Tang C., Mei Q., Sandig G., Rodrigues D. A., Rushmore T. H., Shou M. (2001). Drug Metab. Dispos..

[cit40] Weber P. C., Ohlendorf D. H., Wendoloski J. J., Salemme F. R. (1989). Science.

[cit41] Hendrickson W. A., Pähler A., Smith J. L., Satow Y., Merritt E. A., Phizackerley R. P. (1989). Proc. Natl. Acad. Sci. U. S. A..

[cit42] Bing T., Yang X., Mei H., Cao Z., Shangguan D. (2010). Bioorg. Med. Chem..

[cit43] Ruigrok V. J. B., van Duijn E., Barendregt A., Dyer K., Tainer J. A., Stoltenburg R., Strehlitz B., Levisson M., Smidt H., van der Oost J. (2012). ChemBioChem.

[cit44] Hoinka J., Backofen R., Przytycka T. M. (2018). Mol. Ther. Nucleic Acids.

[cit45] Kurtz S., Narechania A., Stein J. C., Ware D. (2008). BMC Genomics.

[cit46] Kato S., Ono T., Ito M., Ito K., Minagawa H., Horii K., Shiratori I., Waga I., Aoki T. (2021). EAI Endorsed Trans. Bioeng. Bioinform..

