# Supplementary material for: ARCaDia-selected short DNA core positions a latent warhead for protein-gated covalent capture
Source: RSC Chem Biol. 2026 Oct 16;7(11):2183–7. doi: 10.1039/d6cb00303f (PMC13673413; doi:10.1039/d6cb00303f)
Supplement: Supplementary file 1 [file CB-007-D6CB00303F-s001.pdf]

Supplementary Information (SI) for RSC Chemical Biology.
This journal is © The Royal Society of Chemistry

*Supplementary information for:*

# ARCaDia-selected short DNA core positions a latent warhead for protein-gated covalent capture

**Chaohui Li, Naoyuki Miyashita, Masayasu Kuwahara, Jay Yang,* and Masumi Taki***

**Department of Engineering Science, Bioscience and Technology Program, The Graduate School of Informatics and Engineering, The University of Electro-Communications (UEC), 1-5-1 Chofugaoka, Chofu, Tokyo 182-8585, Japan.*

Tel: +81-42-443-5980; E-mail: taki@pc.uec.ac.jp (M.T.), jyang75@wisc.edu (J.Y.)

# Contents:

**1. General**
**2. Synthesis of covalent-binding DNA library**
**3. Screening of the library against the SA target**
3.1. Protein immobilisation on magnetic nanobeads
3.2. Selection against SA-immobilised nanobeads using the AFS-functionalised DNA library
3.3. DNA release from the beads and PCR amplification
3.4. High-throughput sequencing
**4. Analysis of the HTS data**
4.1. Preprocessing and cleanup of the raw HTS reads
4.2. K-mer distribution analysis
4.3. pFSBC analysis
4.4. Alignment of FSBC seeds or HTS reads containing the seeds
**5. PAGE-based validation**
5.1. Summary of PAGE-based assay conditions
5.2. Preparation of warhead-modified Core and Rev oligonucleotides
5.3. SDS-PAGE analysis of Core-AFS conjugation to SA
5.4. Nuclease resistance assay
5.5. Kinetic analysis of Core-AFS covalent binding to SA
5.6. Biotin competition assay
5.7. Parallel native PAGE and SDS-PAGE analysis
5.8. Comparison of AFS, meBSF, and BSF warheads
**6. Statistics**
**7. Supplementary figures**
**8. Uncropped and unprocessed full-gel source images**

## 1. General

All the reagents and solvents were purchased commercially and used without further purification. Streptavidin (SA; Fujifilm Wako Corp, #192-17864, Japan), human α-thrombin (Haematologic Tech Inc, #HCT-0020, USA), human serum (Sigma-Aldrich, #H4522, USA), NeutrAvidin (Thermo Scientific, #31000, USA), and lysozyme (Fujifilm Wako Corp, #123-06721, Japan) were used as received. The Core oligonucleotide containing an octadiynyl-spacer-modified uridine (5-octadiynyl-dU; OdU) in place of a thymine residue, and its complementary strand (CS) were synthesised by Integrated DNA Technologies (IDT) Inc. (Table S1).

**Table S1.** Nucleic acids purchased from IDT.

| Sample name | Oligonucleotide |
|---|---|
| alkyne-containing Core | 5′-GCTTCGA–OdU–GC-3′ |
| alkyne-containing Reverse-order ($Rev_3$) | 5′-CG–OdU–AGCTTCG-3′ |
| alkyne-containing Reverse-order ($Rev_8$) | 5′-CGTAGCT–OdU–CG-3′ |
| alkyne-containing TBA ($TBA_3$) | 5′-GG–OdU–TGGTGTGGTTGG-3′ |
| Core CS | 5′-GCATCGAAGC-3′ |
| TBA CS | 5′-CCAACCACACCAACC-3′ |

Micro-quantitative analyses of oligonucleotides were performed using a reversed-phase semi-micro high-performance liquid chromatography (HPLC) system (PU-2085 with C18 column, JASCO Corp., Tokyo, Japan) connected sequentially to a fluorescence detector and a photodiode array (PDA) detector. Matrix-assisted laser desorption/ionization time-of-flight mass spectrometry (MALDI-TOF MS) analyses were performed on a SpiralTOF mass spectrometer (JMS-S3000, JEOL). All images of stained gels and in-gel fluorescence were captured using a ChemiDoc XRS+ system (Bio-Rad Laboratories Inc., USA) and band intensities were quantified using Image Lab software (Bio-Rad Laboratories Inc., USA).

## 2. Synthesis of covalent-binding DNA library

The covalent-binding DNA library was prepared by conjugating an azide-functionalised sulfur(VI) fluoride exchange (SuFEx) warhead to an alkyne-modified DNA library via copper(I)-catalysed azide–alkyne cycloaddition (CuAAC) as shown below. The azide-functionalised SuFEx warheads used in this study, $N_3$-AFS,[17] $N_3$-meBSF,[23] and $N_3$-BSF,[20] were synthesised, purified according to the reported procedures. Their structural characterisation, including NMR analysis, has been reported previously. For meBSF, the reported azidation was performed at room temperature for 60 min. For ARCaDia selection, the AFS-functionalised DNA library

was used, whereas meBSF and BSF were used for comparative evaluation of warhead-dependent covalent capture.

A DNA library with the sequence 5′-GGGAGGACGATGCGG-ACGT-$N_{12}$-OdU-$N_{12}$-TGCA-CAGACGACGAGCGGGA-3′ (each $N_{12}$ represents a 12-nt randomised region hand-mixed with an equimolar mixture of A, C, G, and T phosphoramidites) was synthesised by Nihon Gene Research Laboratories (NGRL) Inc. The equimolar distribution of the nucleobases within these $N_{12}$ regions has been thoroughly verified by NGS analysis in our previous work utilising the same library.[17] Equimolar amounts (2.5 nmol) of the alkyne-modified DNA library and two CS oligonucleotides targeting the 5′- and 3′-terminal constant/primer regions, 5′-CCGCATCGTCCTCCC-3′, and 5′-TCCCGCTCGTCGTCTG-3′, respectively, were mixed in D-PBS in a total volume of 50 μL. The mixture was heated for 2 min at 95 °C and then cooled to room temperature over 1 h to form the terminal double-stranded regions (underlined). Separately, tris(3-hydroxypropyltriazolylmethyl)amine (THPTA in water, 0.12 μmol, Aldrich # 762342, USA), copper(II) sulfate ($CuSO_4$ in water, 0.03 μmol, Aldrich # 451657, USA), and freshly prepared ascorbic acid (neutralised by NaOH in water, 0.75 μmol, Aldrich # A92902, USA) were successively mixed in water to a total volume of 30 μL, and incubated at room temperature for 10 min to prepare the CuAAC catalyst solution. Then, the annealed alkyne-containing library solution (2.5 nmol, 50 μL), azidated warhead (in DMSO, 0.05 μmol, 2 μL), and the pre-incubated catalyst solution (7.5 μL) were combined, and the mixture was reacted for 1 h at 4 °C. The crude reaction product was purified by ethanol precipitation. To the crude product, sodium acetate solution (3 M, pH 5.2, 0.1 volumes relative to the reaction mixture) and pre-cooled ethanol were added. After centrifugation (15,000 rpm, 40 min, 4 °C), the supernatant was removed, and the pellet was washed with 70% ethanol. The residue was dissolved in PBS supplemented with KCl (15 mM), to obtain the warhead-functionalised DNA library at 33.8 μM. As a negative control, benzyl azide (Wako #357-40761) was used instead of the azidated warhead to obtain the corresponding 'non-reactive' library.

## 3. Screening of the library against the SA target

### 3.1. Protein immobilisation on magnetic nanobeads

Proteins, either SA as the target or bovine serum albumin (BSA) as a target-unrelated control, were covalently immobilised on magnetic nanobeads (FG-NHS Beads, Tamagawa Seiki #TAS8848N1141, Japan) using a Protein Immobilisation Buffer Kit (#TAB1200N0319) according to the manufacturer's protocol. Briefly, methanol-washed NHS beads (1 mg, ca. 0.25 μmol functional groups) were suspended in 50 μL of HEPES-NaOH reaction buffer (25 mM, pH 7.0), and briefly sonicated. Then, 50 μg of protein (*e.g.*, SA, 0.94 nmol as tetramer) in the same buffer (50 μL) was added to the bead suspension, and the mixture was incubated overnight at 4 °C on a rotator, followed by removal of the supernatant. After the immobilised protein was indirectly quantified from the supernatant by the Bradford method using

Quick Start Bradford Reagent (1× kit; Bio-Rad), the unreacted NHS functional groups on the beads were blocked using 0.25 mL of 2-aminoethanol (1.0 M, pH 8.0) containing 0.1% (w/v) NP-40. The suspension was incubated overnight at 4 °C on a rotator, followed by removal of the supernatant. The beads were washed three times with Wash/Storage Buffer containing 10 mM HEPES-NaOH (pH 7.9), 50 mM KCl, 1 mM EDTA, and 10% (v/v) glycerol at 4 °C. The protein-immobilised beads were stored in the same buffer in a refrigerator. Approximately 42 µg (0.79 nmol) of SA and 33 µg (0.50 nmol) of BSA were immobilised per 1 mg of beads, which were suspended in the buffer (0.20 mL). Just before biopanning, 10 µL (*i.e.*, 50 µg of beads per a single batch) of the suspension was pre-washed with D-PBS (0.20 mL), and the supernatant was removed.

### 3.2. Selection against SA-immobilised nanobeads using the AFS-functionalised DNA library

Before targeted selection, a negative counter-selection against BSA-immobilised beads was performed using the warhead-functionalised DNA library to remove non-specific binders (*i.e.,* bead-, plastic-, and BSA-binders). The library (14.2 µL; 0.48 nmol, $2.9 \times 10^{14}$ molecules/sample) was pre-incubated with the BSA-conjugated beads (50 µg of beads; 25 pmol BSA/sample) overnight at 37 °C with shaking at 800 rpm using a maximiser (MBR-022UP, Taitec, Saitama, Japan). For the targeted selection, the resulting supernatant was further incubated with the SA-immobilised beads (50 µg; 39.5 pmol SA/sample) overnight at 37 °C. Then, the beads were washed with 0.20 mL of a stringent wash buffer (50 mM Tris-HCl (pH 8.0), 1.0% (w/v) SDS, 4.0 M urea, 0.15 M NaCl) under continuous sonication (30 min), and this washing process was repeated three times. Then, the washing was performed once again at 50 °C (30 min) under occasional sonication with shaking at 800 rpm using the maximiser. The beads were further washed with D-PBS (0.20 mL) twice.

### 3.3. DNA release from the beads and PCR amplification

Candidate DNAs were released from the SA-immobilised nanobeads by digestion with proteinase K (ProK; Sigma, St. Louis, MO, USA). Briefly, 2.5 µL of ProK (1 µg/µL) in digestion buffer (50 mM Tris-HCl (pH 8.0), 10 mM $CaCl_2$) was added, and the mixture was incubated for 1 h at 50 °C with shaking at 800 rpm using the maximizer. Pure water (3.5 µL) was then added. The beads were further incubated overnight at 50 °C without shaking in the dark, and then 0.2 µL of 0.10 M phenylmethanesulfonyl fluoride (PMSF) in ethanol was added to deactivate the enzyme. The mixture was incubated at room temperature for 30 min, and 6.0 µL of 2× PCR buffer for PWO polymerase was added. The released DNA was recovered in the liquid phase by boiling in the buffer at 98 °C (10 min) under occasional sonication with shaking, and 1.0 µL of the liquid (total volume, ca.12 µL) was used as a template for a hot-start polymerase chain reaction (PCR). The PCR was carried out with 12 cycles of denaturation at 95 °C for 30 sec, annealing at 52 °C for 30 sec, and extension at 72 °C for 1 min in a 10 µL reaction using a forward primer (5′-

GGGAGGACGATGCGG-3′, purchased from IDT) and a reverse primer (5′-TCCCGCTCGTCGTCTG-3′, purchased from IDT), and PWO SuperYield DNA polymerase (Sigma, #63832027). Each PCR product was separated by polyacrylamide gel electrophoresis (PAGE), and the gel was stained with SYBR Gold (Thermo Fisher, #3268190) (Fig. S3).

### 3.4. High-throughput sequencing

The amplified selected DNA band (64 bp) was excised from the PAGE gel, eluted into pure water (20 µL) by occasional sonication and boiling, and 1.0 µL of the solution was used as a template for PCR (10 cycles of denaturation at 95 °C for 30 sec, annealing at 58 °C for 30 sec, and extension at 72 °C for 1 min) to further amplify the library-coding region using a forward primer (5′-GAACGACATGGCTACGATCCGACTTTCTGCGCCTTCCTCGTCGGCAGCGGCCAATGGGAGGACGATGCGG-3′), a reverse primer (5′-GTCTTCCTAAGACCGCTTGGCCTCCGACTTTCTGCGCCTTCCTCGTCGGCCAATTCCCGCTCGTCGTCTG-3′), and GoTaq DNA polymerase (Promega). The first PCR product (173 bp) for high-throughput sequencing (HTS) was purified by 12% PAGE followed by ethanol precipitation and submitted to a vendor (Bioengineering Lab. Co., Ltd.) for the HTS analysis using an MGI DNBSEQ-G400; the PCR amplification for preparing the DNBSEQ library was performed by the vendor.

## 4. Analysis of the HTS data

### 4.1. Preprocessing and cleanup of the raw HTS reads

The forward and reverse raw reads in FASTQ format provided by the vendor were imported into AptaSuite (v0.9.8)[44] with the designated forward and reverse primer sequences. Reads with 5′- or 3′-terminal errors, invalid characters, or inconsistency between the two reads were eliminated, leaving the cleaned HTS reads with a 25-nt randomised insert. In total, >90% (1,145,389 accepted reads / 1,267,316 processed reads) of the raw reads were accepted. The HTS reads after cleanup were output as an 'HTS.fasta' file using the Export option for further analysis. Before pFSBC analysis, the FASTA headers exported from AptaSuite were converted into numeric IDs to avoid parsing errors in the pFSBC_custom.py script.

### 4.2. *K*-mer distribution analysis

Tallymer[45] running under Ubuntu Linux (v22.04.3) in Oracle VM VirtualBox on a MINISFORUM UM330-8 computer running Windows 11 was used for the *k*-mer analysis. The most frequent *k*-mer sequence(s) at each *k*-mer length was defined as the 'top seed(s)', and the second most frequent sequence(s) as the 'second seed(s)'. The output of *k*-mer analysis for 7–14-mers is shown in Table S2. A *k*-mer analysis of a negative control library before any screening gave no obvious consensus sequence. Details of the *k*-mer analysis can be found in our previous publication.[17]

**Table S2. Top *k*-mer analysis of 1,145,389 accepted SA-selection HTS reads.** Only the top and second-ranked seed strings (*i.e.,* subsequences) for *k*-mer lengths 7-14 are shown, because the Core-overlapping seed appeared at $k = 13$ and longer *k*-mers were dominated by low-complexity/G-rich extensions without changing the conclusion. In this SA-selection dataset, top *k*-mer analysis preferentially highlighted G-rich/low-complexity seed strings. A second-ranked 13-mer seed overlapped with the pFSBC-identified Core motif, shown in bold and underlined, indicating enrichment around the Core region. However, top *k*-mer analysis alone did not define the compact Core boundary or the modified-nucleotide position. Therefore, pFSBC analysis followed by U*-position mapping was used to assign the compact Core motif as <u>**GCTTCGAU*G**</u>C (see below).

| *k*-mer length | Top count | Top seed(s) | Second count | Second seed(s) |
|---:|---:|---|---:|---|
| 7 | 8704 | GGGGGGT | 8209 | TGGGGGG |
| 8 | 2473 | GGGGGGTT | 2442 | GGGGGGTG |
| 9 | 725 | GGGGGGTGT | 712 | GTGGGGGGT |
| 10 | 219 | GGGGGGTGTT | 205 | GGTGGGGGGT |
| 11 | 72 | GGGGGGGTGTT | 68 | GGGGGGTTGTT |
| 12 | 28 | GGGGGGGGGGGG | 26 | GGGGGGTTGTTT;<br>GGGGGGTTGGTT |
| 13 | 18 | GGGGGGGGGGGGG | 14 | GG<u>**GCTTCGATG**</u>GC |
| 14 | 14 | GGGGGGGGGGGGGG | 9 | GGGTGATGGTATGG;<br>GGGGTTAGATGCCC |

### 4.3. pFSBC analysis

The cleaned HTS reads were analysed using pFSBC,[46] a Python implementation of fast string-based clustering (FSBC).[34] For the present $N_{12}$–U*–$N_{12}$ library, the AFS-modified uridine analogue at the fixed central position was read as T in the sequencing output; therefore, the pFSBC analysis was performed with the standard DNA alphabet A, C, G, and T. The U* position was assigned later by mapping the fixed 13$^{th}$ nucleotide onto the aligned seed-containing reads, as described below.

The pFSBC analysis was performed locally using an updated script, pFSBC_custom.py, provided as part of the SI. This script was modified for compatibility with current Python/Pandas versions without changing the original FSBC algorithm or scoring procedure. pFSBC calculates the empirical nucleotide composition of the read set and then evaluates the enrichment of each string against the expected occurrence probability calculated from this nucleotide composition. For a given string `s`, the observed frequency ($F_s$) of reads containing `s` is compared with the expected probability of observing `s` in the read set ($P_s$), and a $Z_s$-score is calculated. To compare strings of different lengths, the $Z^*_s$-score was further

normalised within each string length class to give a length-adjusted score, which was used for ranking the extracted strings.[46]

The initial pFSBC search was generally performed with string lengths of 7–12 nucleotides, because this range provided rapid extraction of short, locally enriched motifs while avoiding excessive noise from very short strings. When the highest-ranked strings appeared to be extendable, or when the alignment of the extracted reads suggested a longer conserved region, the maximum string length was increased stepwise to 15 nucleotides. Strings were extended only when the extended string improved the enrichment score relative to the shorter parent string, following the search-space-reduction logic of pFSBC. The final analysis was executed locally using this modified version, and the ranked strings along with their corresponding full-length read sets were used for subsequent alignment and candidate identification. Candidate motifs were not selected solely by full-length read abundance. Instead, the ranking of pFSBC strings, the consistency of the multiple sequence alignment, the local conservation of the motif, and the positional relationship between the motif and the fixed AFS-modified nucleotide were evaluated together.

In the present SA selection, this workflow identified a conserved short core motif in which the modified nucleotide could be assigned within the aligned local sequence, yielding the U*-containing core used for subsequent chemical synthesis and validation. To assign the position of the AFS-modified nucleotide within the pFSBC-identified seed, full-length 25-nt HTS reads containing the seed sequence GCTTCGATGC were extracted and aligned. In the starting $N_{12}$–U*–$N_{12}$ library, the modified nucleotide was fixed at the 13th nucleotide of the 25-nt variable region and was read as T in the sequencing output. Therefore, for each aligned read, the 13th nucleotide position was mapped onto the corresponding position within the seed sequence. Among the seed-containing reads, the fixed 13th nucleotide most frequently corresponded to the 8th position of the seed (Table S3). Accordingly, the pFSBC seed GCTTCGATGC was assigned as GCTTCGAU*GC and was chemically synthesised as Core-AFS for validation experiments. pFSBC analysis of the unselected library did not reveal a reproducible high-frequency consensus motif.

**Table S3. Positional assignment of U* within the pFSBC top seed GC$\underline{T}_3\underline{T}_4$CGA$\underline{T}_8$GC.**

| T position in seed | Read count | Fraction |
|---|---|---|
| 3 | 9 | 8.4% |
| 4 | 6 | 5.6% |
| **8** | **79** | **74%** |
| outside | 13 | 12% |

## 4.4. Alignment of HTS reads containing pFSBC-identified seed sequences

Full-length HTS reads containing the pFSBC-identified seed sequences were extracted using the Search function of AptaSuite. The extracted sequences and their

ID tags were exported as a tab-delimited text file, converted to FASTA format using Tab to Fasta Converter (bugaco.com), and aligned using CLUSTALW (genome.jp). The aligned '*.aln' file was visualised using Jalview software, and the consensus sequence with a threshold of >50% was displayed with a black-shaded background, where the intensity of the black shading was proportional to the conservation score of each column in the alignment.

## 5. PAGE-based validation

### 5.1. Summary of PAGE-based assay conditions

The experimental conditions for the PAGE-based validation assays are summarised in Table S4 before the individual procedures are described below.

**Table S4. Summary of assay conditions for PAGE-based validation.**

| Assay (PAGE) | Target protein | Oligonucleotide | Additive or treatment | Incubation |
|---|---|---|---|---|
| Nuclease resistance (SDS-P; Section 5.4) | SA, 10 µM; thrombin, 5.5 µM | Core-AFS, 90 µM; $TBA_3$-BSF, 22 µM | 10-fold CS, followed by DNase I (10 U) or Benzonase (2.5 U) | Conjugation: 37 °C, 24 h; hybridisation: 20 °C, 1 h; digestion: 20 °C, 24 h |
| Conjugation kinetics (SDS-P; Section 5.5) | SA, 6.7 µM | Core-AFS Varying: 1.7–347 µM | None | 37 °C; 1–96 h |
| Biotin competition (SDS-P; Section 5.6) | SA, 8.6 µM | Core-AFS or Rev-AFS, 39 µM | Biotin, 69 µM; added before or after oligonucleotide incubation | 37 °C, 46 h |
| Parallel PAGE (native / SDS-P; Section 5.7) | SA, 6.7 µM | Core-AFS or Rev-AFS, 37 µM | Biotin, 54 µM, where indicated | 37 °C, 96 h |
| Warhead comparison (SDS-P; Section 5.8) | SA, 10 µM | Core- or Rev-AFS, -meBSF or -BSF, 45 µM | None | 37 °C, 24 h |

The reaction buffer was 1× PBS supplemented with 15 mM KCl and 2 mM $MgCl_2$. Unless otherwise stated, values in the table represent final concentrations. For the nuclease resistance assay, the listed protein and oligonucleotide concentrations indicate the concentrations in the primary conjugation mixtures before the addition of CS and nucleases. In the biotin competition assay, biotin was used at a two-fold molar excess relative to SA monomer. 'Added before' indicates pre-incubation of SA with biotin for 20 min at room temperature before addition of the

oligonucleotide, whereas 'added after' indicates the addition of biotin after oligonucleotide incubation. Samples without biotin were supplemented with an equivalent volume of reaction buffer. Unless otherwise stated, SDS-PAGE samples were mixed with 4× SDS-PAGE loading buffer containing 2-mercaptoethanol, heated at 95 °C for 10 min, and analysed using 13% polyacrylamide gels. Electrophoresis was carried out at 80 V for 20 min and then at 120 V for 80 min.

## 5.2. Preparation of warhead-modified Core and Rev oligonucleotides

AFS-modified Core and Rev oligonucleotides were prepared by CuAAC conjugation of the corresponding alkyne-modified oligonucleotides with the azide-functionalised AFS warhead. A catalyst solution was prepared by sequentially mixing THPTA (40 nmol), $CuSO_4$ (10 nmol), and sodium ascorbate (0.25 μmol) in water and incubating the mixture at room temperature for 10 min. The alkyne-modified oligonucleotide (10 nmol), azide-functionalised warhead (25 nmol in DMSO), and the pre-incubated catalyst solution were then combined, and water was added to give a final volume of 25 μL. The reaction mixture was incubated for 1 h at 4 °C.

The crude reaction mixture was purified by ethanol precipitation. Sodium acetate solution (3 M, pH 5.2, 2.5 μL; 0.1 volumes relative to the reaction mixture) and pre-cooled ethanol (55 μL) were added to the reaction mixture, and the sample was centrifuged. The supernatant was removed, and the pellet was washed with 70% ethanol. The pellet was briefly dried and dissolved in 20 μL of reaction buffer. The concentration of the warhead-functionalised oligonucleotide was estimated from UV absorbance using a NanoPhotometer. The same procedure was used to prepare meBSF- and BSF-modified oligonucleotides, where indicated.

The purity of the purchased Core-OdU and Rev-OdU before and after the AFS-warhead modification was examined by HPLC (Fig. S1) and the mass shift supporting successful modification was directly measured using MALDI-TOF MS (Fig. S2).

HPLC separation was carried out using a linear gradient of 0–60% acetonitrile in 20 mM aqueous triethylammonium acetate (TEAA) solution (pH 7.4; prepared from TEAA, 2.0 mol/L in water for HPLC, Product Code: T4022, Tokyo Chemical Industry Co., Ltd., Tokyo, Japan) over 26 min at a flow rate of 200 μL/min. For MALDI-TOF MS, 3,4-diaminobenzophenone (DABP; Product Code: D1950, Tokyo Chemical Industry) was used as the matrix, prepared at a concentration of 15 mg/mL in a solvent mixture of methanol and aqueous hydrochloric acid (80:3, v/v). The oligonucleotide samples were mixed with the DABP matrix solution at a 1:1 (v/v) ratio, and 0.5 μL of the mixture was spotted onto a stainless steel MALDI target plate and allowed to air-dry at room temperature. The spectra were obtained in spiral negative-ion mode, and all spectra were externally calibrated using an 8-nt reference oligonucleotide (5′-GCGTAAGC-3′) based on its theoretical monoisotopic $[M - H]^-$ peak at $m/z$ 2432.4485.

### 5.3. SDS-PAGE analysis of Core-AFS conjugation to SA

Covalent conjugation of Core-AFS to SA was evaluated by reducing SDS-PAGE. Core-AFS or Rev-AFS was mixed with SA in reaction buffer and incubated at 37 °C for the indicated times. The samples were separated by SDS-PAGE, and the protein bands were visualised with Coomassie Brilliant Blue (CBB, Nacalai, #M4F5573) staining or Oriole Fluorescent Protein Gel Stain (Bio-Rad, #1610496).

The specific assay conditions were as follows: For the serum-containing assay, Core-AFS or Rev-AFS was incubated with SA in the presence of 5% human serum. For the target-selectivity assay, Core-AFS or Rev-AFS was incubated with SA, NeutrAvidin, or lysozyme under otherwise identical conditions. For the concentration-dependence assay, the reaction mixture was adjusted with reaction buffer, and SA was incubated with increasing concentrations of Core-AFS for 24 h at 37 °C. Band intensities were quantified as described above. The extent of covalent conjugation was calculated as the intensity of the mobility-shifted SA band divided by the sum of the shifted and unshifted SA bands.

### 5.4. Nuclease resistance assay

To evaluate the nuclease resistance of Core-AFS after covalent conjugation to SA, the Core-AFS-SA conjugate was treated with nucleases, in parallel with the $TBA_3$-BSF-thrombin conjugate as a positive control, as reported previously.[22] For the primary covalent labelling reaction, 3.33 μL of a 20 μM SA solution was mixed with 3.33 μL of a 180 μM Core-AFS solution. In parallel, 5.0 μL of an 11 μM thrombin solution was mixed with 5.0 μL of a 44 μM $TBA_3$-BSF solution. Both reaction mixtures were incubated at 37 °C for 24 h to allow covalent bond formation. Following conjugation, a 10-fold molar excess of the corresponding CS relative to each warhead-modified oligonucleotide was added from a 1 mM aqueous CS stock solution (6.0 μL Core CS or 0.55 μL TBA CS), and hybridisation was performed at 20 °C for 1 h. The 20 °C incubation was used to promote duplex formation between the short 10-nt Core-AFS and its CS. Enzymatic digestion was initiated by adding either 10 U of DNase I with DNase I buffer at a final 1× concentration (Biolabs, Cat. No. 10105300) or 2.5 U of Benzonase alone (Biolabs, Cat. No. SLCP0995), and the reactions were maintained at 20 °C for 24 h. After addition of 4× SDS-PAGE loading buffer containing 2-mercaptoethanol to achieve a 1× final concentration, the samples were boiled at 95 °C for 5 min, placed on a 4 °C cooling block, briefly centrifuged, carefully loaded into the gel wells at an ambient temperature of 15 °C, and separated by SDS-PAGE at 4 °C (Fig. S4).

### 5.5. Kinetic analysis of Core-AFS covalent binding to SA

The covalent conjugation kinetics of Core-AFS toward SA were quantified using time-course SDS-PAGE band-shift ratio analysis. Briefly, the reaction was conducted in a total volume of 10 μL by mixing 3.33 μL of SA tetramer (20 μM stock, yielding a final concentration of 6.7 μM) with 3.33 μL of Core-AFS at varying stock concentrations to give final reaction concentrations of 1.7, 5.0, 10, 20, 30, 37,

50, 77, 90, 145, 200, and 347 μM. The remaining volume was adjusted with 3.33 μL of reaction buffer. The mixtures were incubated at 37 °C for designated time intervals (1, 4, 8, 12, 24, 72, and 96 h). At each time point, the reaction was terminated by adding 3.33 μL of 4× SDS-PAGE loading buffer (achieving a 1× final concentration) followed by boiling at 95 °C for 10 min. The samples were resolved by SDS-PAGE. The gel was sequentially stained with Oriole Fluorescent Protein Gel Stain for sensitive protein detection and then with CBB. Band intensities were quantified as described above to determine the ratio of the mobility-shifted covalent adduct relative to the total SA band intensity.

The time-course data obtained at each Core-AFS concentration were analysed using GraphPad Prism 6 (GraphPad Software, San Diego, CA, USA). The data were fitted to a mono-exponential association equation: $f(t) = f_\infty(1 - e^{-k_{obs} \cdot t})$ where $f(t)$ is the fractional conjugation at time $t$, $f_\infty$ is the plateau amplitude, and $k_{obs}$ is the apparent first-order rate constant. The obtained $k_{obs}$ values were plotted against the corresponding concentrations of Core-AFS and fitted to a phenomenological partial-substrate-inhibition model (Fig. 3). As an alternative description, the same data were also fitted to a dimer-competition model (Fig. S5). The kinetic equations for $k_{obs}$ and the numerical values of the best-fit model parameters are shown in the insets of the respective figures.

**5.6. Biotin competition assay**

Biotin competition experiments were performed to examine whether Core-AFS conjugation is associated with, or occurs in close functional proximity to, the canonical biotin-binding pocket of SA. SA was reacted with either Core-AFS or Rev-AFS in reaction buffer. In the 'added before' condition, SA was pre-incubated with a two-fold molar excess of free biotin (0.5 mM stock solution; Tokyo Chemical Industry; #B0463) at room temperature for 20 min before the addition of the AFS-modified oligonucleotide. In the 'added after' condition, SA was first incubated with the AFS-modified oligonucleotide, followed by the addition of free biotin and a further 20 min incubation at room temperature (see Table S4 for details). Control reactions lacking biotin or oligonucleotide were volume-matched using the same buffer. The covalent reaction was carried out at 37 °C for 46 h and analysed by reducing SDS-PAGE (Fig. S6).

**5.7. Parallel native PAGE and SDS-PAGE analysis**

Parallel native PAGE and SDS-PAGE analyses were performed using identical reaction mixtures to compare non-denaturing complex formation with SDS-resistant covalent adduct formation. For each test group, Core-AFS or Rev-AFS was reacted with SA in a total volume of 20 μL, either in the absence of biotin or after biotin pre-incubation (Fig. 4A).

For the biotin-pretreated samples, SA was initially incubated with a two-fold molar excess of free biotin relative to SA monomer at room temperature for 20 min

before the addition of the oligonucleotide. The reaction mixtures were incubated at 37 °C for 96 h to allow the SDS-PAGE band-shift ratio to approach its plateau.

Following the 96-h incubation, each reaction mixture was divided into two equal aliquots (10 µL each) for parallel native and denaturing analyses:

**Native PAGE aliquot:** The first half was mixed with a native DNA loading buffer and directly resolved on an 8% native PAGE gel prepared in 0.5× TBE (Tris-borate-EDTA, pH 8.3, Takara, #T9122) containing 3 mM $MgCl_2$. The running buffer was 0.5× TBE supplemented with 2 mM $MgCl_2$. Electrophoresis was conducted at 75 V for 80 min. The gel was sequentially stained with SYBR Gold, Oriole Fluorescent Protein Gel Stain, and CBB.

**SDS-PAGE aliquot:** The second half was mixed with SDS-PAGE loading buffer and heated as described above. The denatured samples were resolved via SDS-PAGE and sequentially stained with SYBR Gold, Oriole Fluorescent Protein Gel Stain, and CBB. Final concentrations are summarised in Table S4 above.

### 5.8. Comparison of AFS, meBSF, and BSF warheads

To compare the effect of warhead structure on covalent conjugation, Core and Rev oligonucleotides modified with AFS, meBSF, or BSF were prepared by CuAAC as described in Section 5.2. Each warhead-modified oligonucleotide (final concentration, 45 µM) was reacted with SA (final concentration, 10 µM) in reaction buffer at 37 °C for 24 h. The samples were analysed by reducing SDS-PAGE.

## 6. Statistical calculations

Unless otherwise stated, PAGE-based validation experiments were repeated at least three times, and the data are presented as mean ± SEM where appropriate. For kinetic parameter determinations (e.g., Fig. 3), assays were performed in duplicate ($n = 2$).

## 7. Supplementary figures

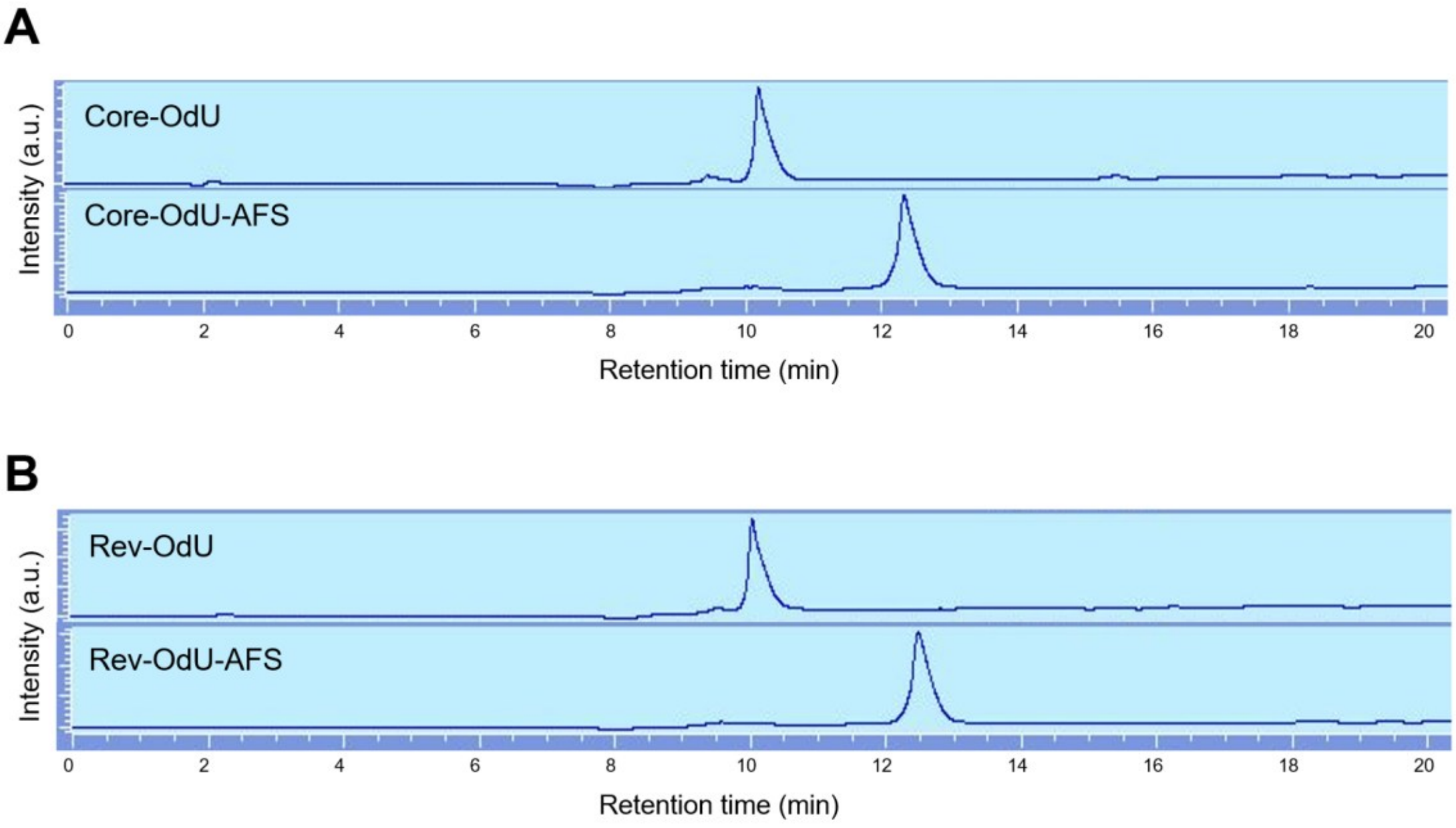


**Fig. S1.** HPLC separation confirms quantitative AFS conjugation. 300 pmol of Core-OdU (A) or Rev-OdU (B) prior to and after AFS conjugation were separated by HPLC and monitored at 260 nm as described in Section 5.2. The purchased oligonucleotides gave a single peak with a retention time near 10 min, which shifted to 12.5 min after the conjugation. The post-modification chromatograms showed essentially complete conversion, with no appreciable residual unmodified oligonucleotides.

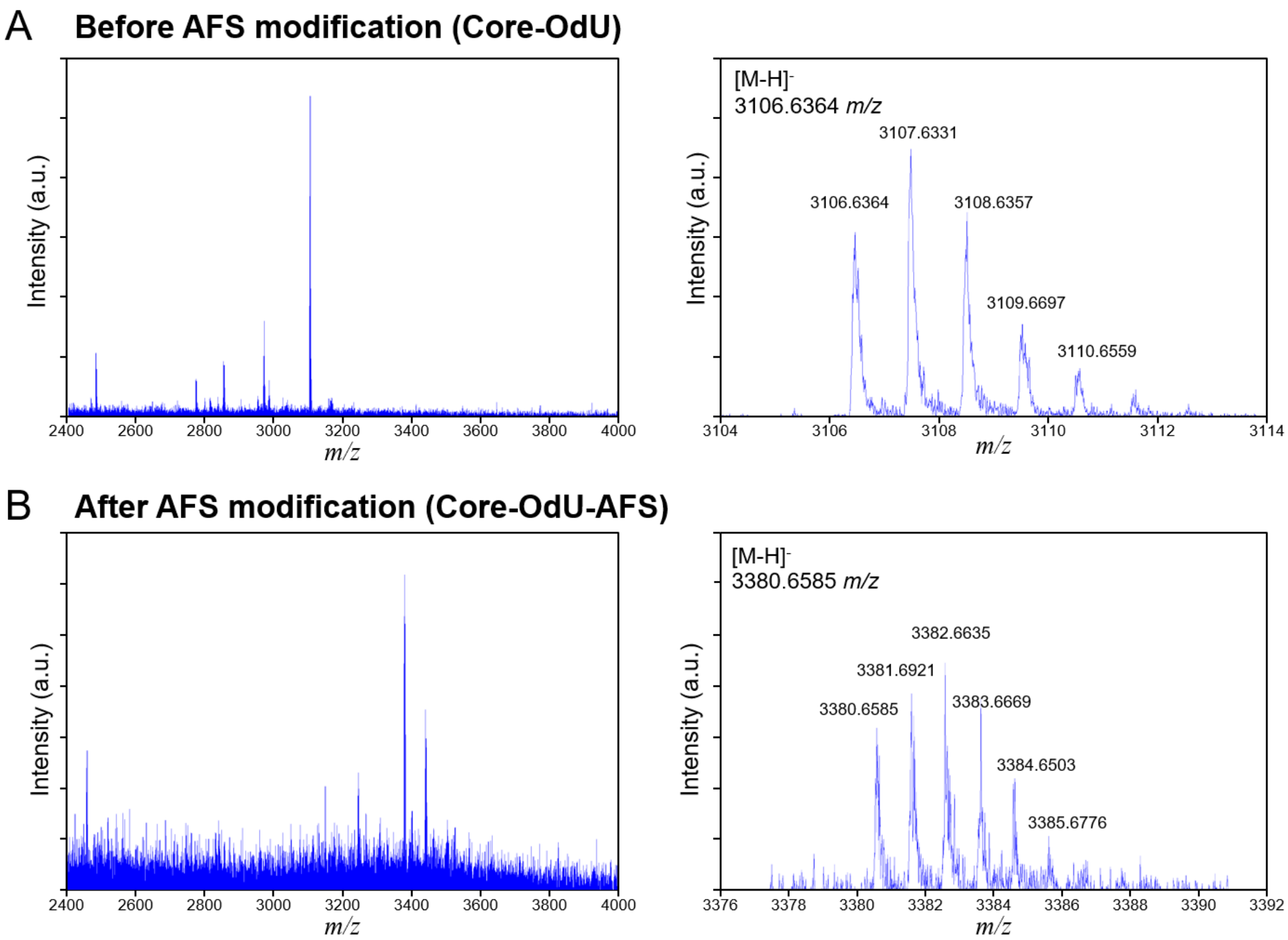


**Fig. S2.** MALDI-TOF spectra of Core oligonucleotides. The scans show the complete acquired spectra from *m/z* 2400–4000 for 25 pmol of Core-OdU before (A) and after (B) AFS modification. The insets show the enlarged peaks with clearly distinguishable isotopic peaks. Comparison of the $[M-H]^-$ peaks shows a mass shift of 274.02 Da which is consistent with successful conjugation of the $N_3$-AFS moiety.

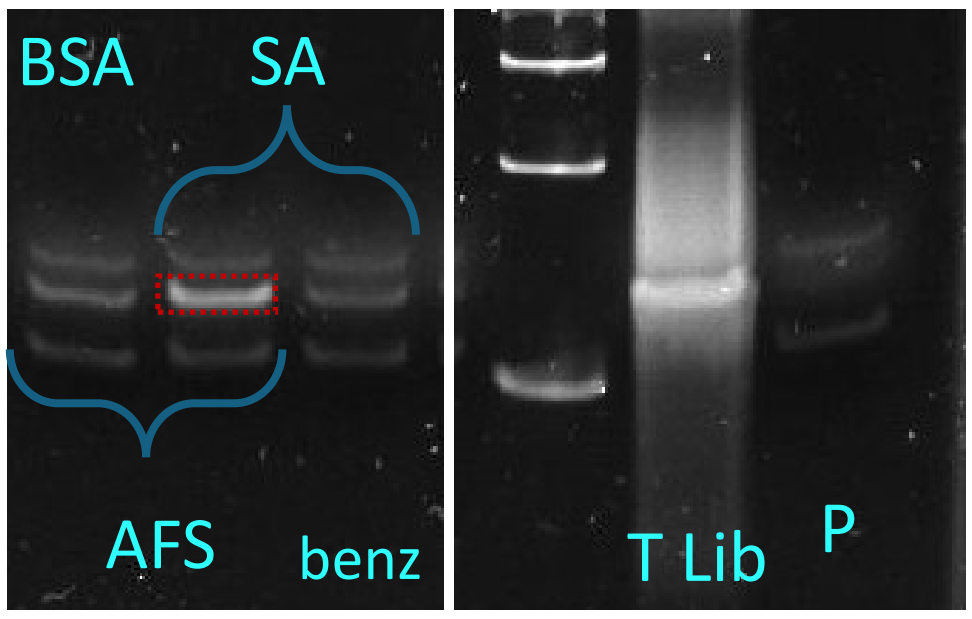


**Fig. S3.** PAGE analysis of PCR-amplified bands after single-round ARCaDia selection against SA. The $N_{12}$–OdU–$N_{12}$ library was conjugated with AFS or benzyl azide (benz) as a non-reactive control and subjected to selection against SA-immobilised beads after BSA-bead counter-selection. After stringent washing and ProK digestion, recovered DNA was amplified by PCR using PWO polymerase for 12 cycles. The expected size of the amplified product is 64 bp, as confirmed by direct PCR amplification of the starting library (T Lib). The DNA size marker bands are 150, 100, and 50 bp. P indicates the primer-only negative control. The 64 bp PCR product from the AFS-selected SA sample was excised (indicated by the red dashed line), re-amplified, and submitted for HTS analysis as described in Section 3.4.

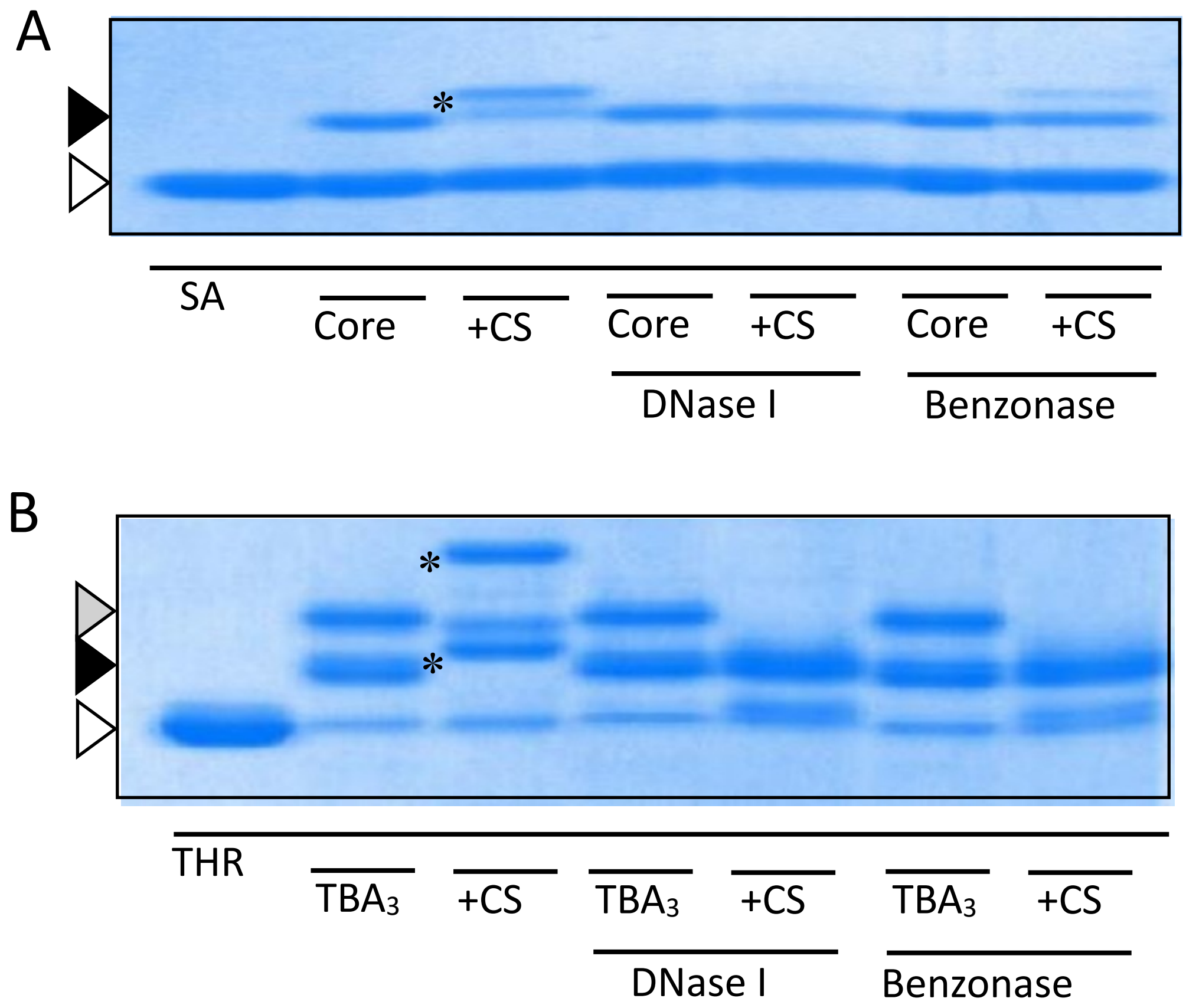


**Fig. S4.** Relative nuclease resistance of Core-AFS covalently conjugated to SA. (A) Denaturing SDS-PAGE of the Core-AFS conjugate in the absence or presence of the complementary strand (CS). Open arrow indicates unreacted SA and closed arrow indicates the mobility-shifted Core-AFS-SA covalent adduct. Addition of CS causes a further upward mobility shift (*). Digestion with DNase I or Benzonase eliminated the CS-dependent shift (*), whereas the Core-AFS conjugated band remained intact indicating nuclease resistance. (B) A control experiment with thrombin (THR) and $TBA_3$-BSF with or without its CS showed nuclease resistance of the covalently bound DNA consistent with our previous report.[22] In panel B, the open, closed, and grey open arrows indicate unreacted THR, the mobility-shifted $TBA_3$-BSF-THR covalent adduct, and a putative bis-adduct containing two $TBA_3$-BSF molecules conjugated to one thrombin molecule, respectively. Note that hybridisation and nuclease digestion were conducted at 20 °C to enable double-strand formation between the Core-AFS and CS. The thrombin target experiment in B was also conducted at 20 °C to confirm sufficient activity of the nucleases although the longer TBA and its CS annealed at a higher temperature. Reaction conditions are described in Table S4 'Nuclease Resistance', and Section 5.4. Panels A and B were analysed on the same SDS-PAGE gel under identical staining conditions.

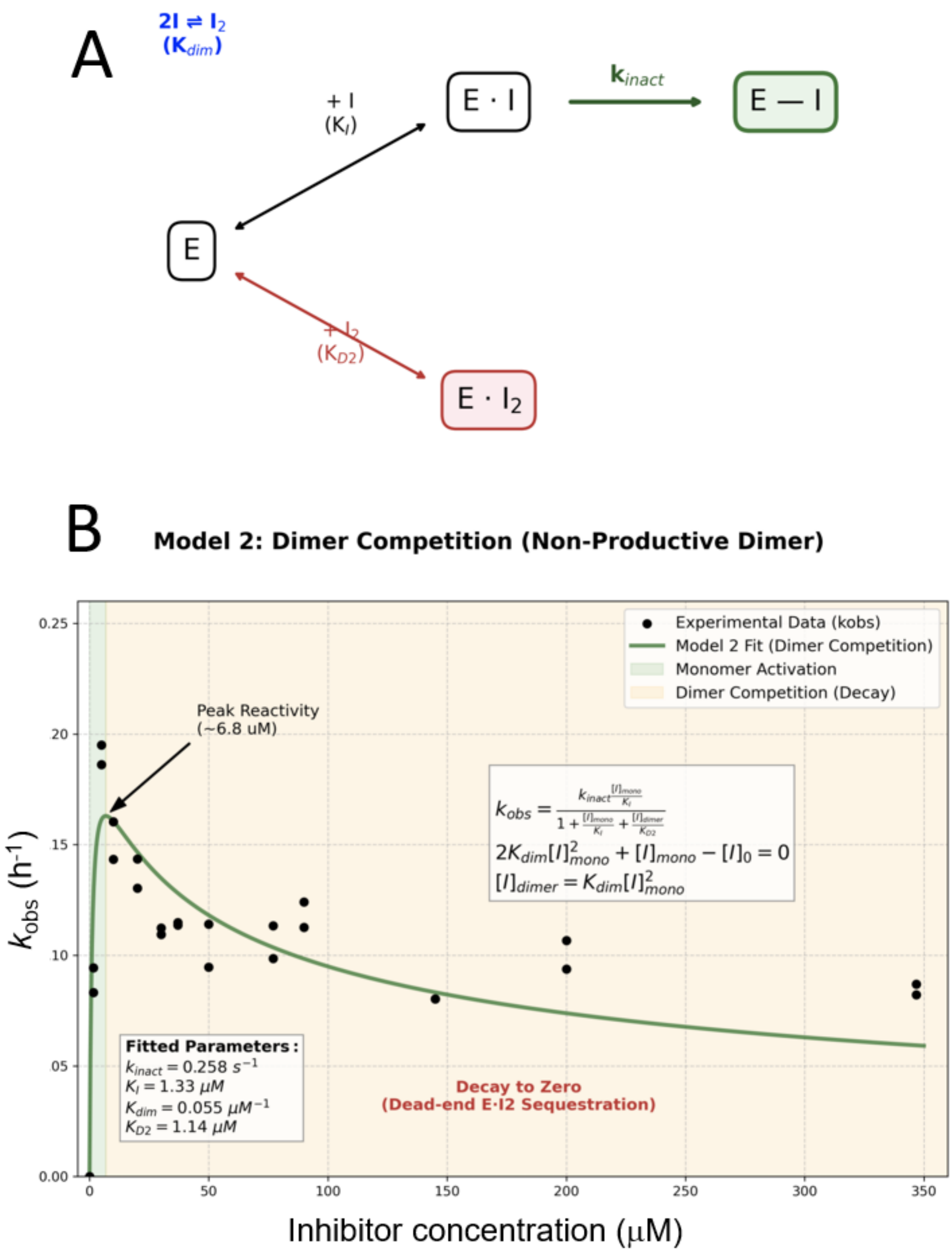


**Fig. S5.** Alternative dimer-competition model to describe the dependence of $k_{obs}$ on Core-AFS concentration. (A) A compartmental model of dimer competition involving a non-productive dimer. (B) Experimental data points (dots) and the model fit are shown. The kinetic equation and the fitted parameters are shown as insets.

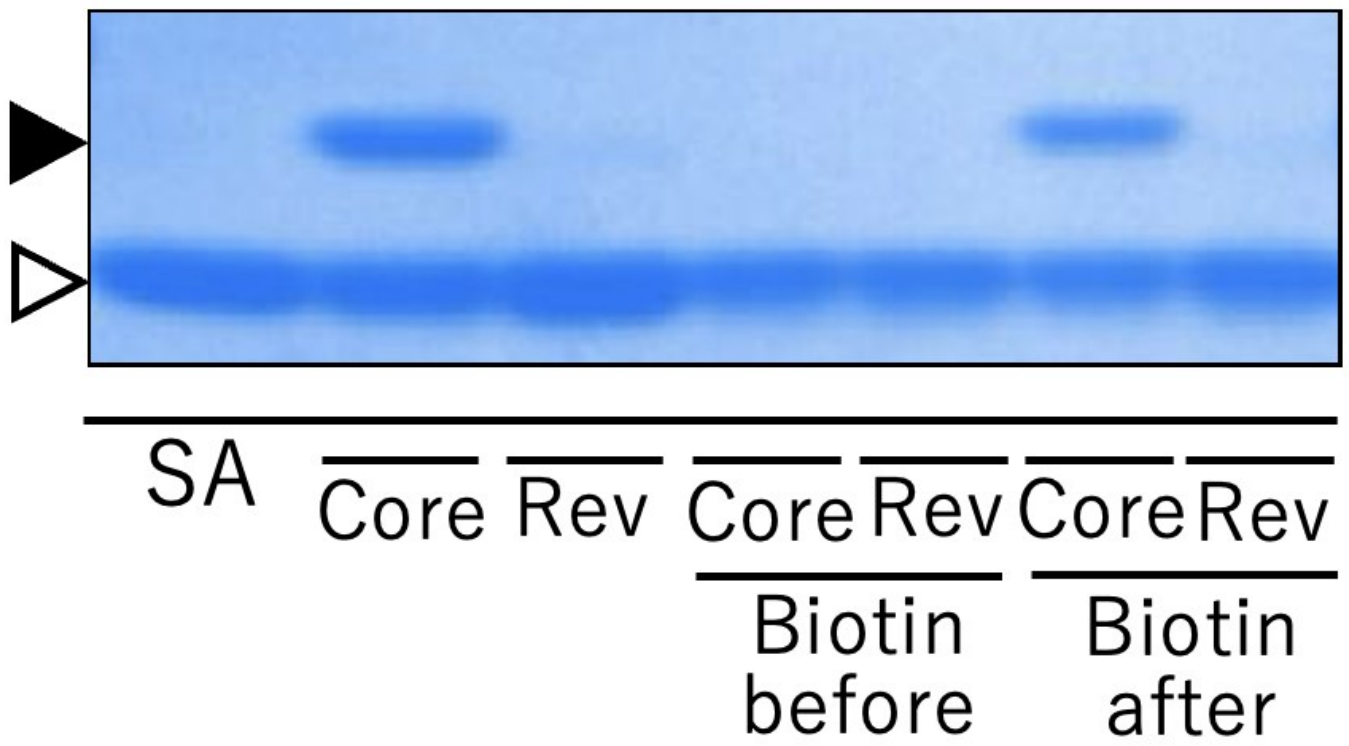


**Fig. S6.** Reducing SDS-PAGE analysis of biotin competition assay for Core-AFS and Rev-AFS conjugation to SA. Core-AFS or Rev-AFS was incubated with SA in the absence or presence of free biotin. In the 'Biotin before' condition, SA was pre-incubated with free biotin before the addition of the AFS-modified oligonucleotide. In the 'Biotin-after' condition, free biotin was added after the conjugation reaction. The samples were analysed by reducing SDS-PAGE. Open and closed arrows indicate unmodified SA and the mobility-shifted covalent adduct, respectively. Reaction conditions are described in Table S4, 'Biotin competition', and Section 5.6.

## 8. Uncropped and unprocessed full-gel source images

Uncropped and unprocessed full-gel source images corresponding to the electrophoresis panels in the main article and Supplementary Information are provided below. The figure references identify the corresponding displayed panels.

### Source image corresponding to Fig. 2B

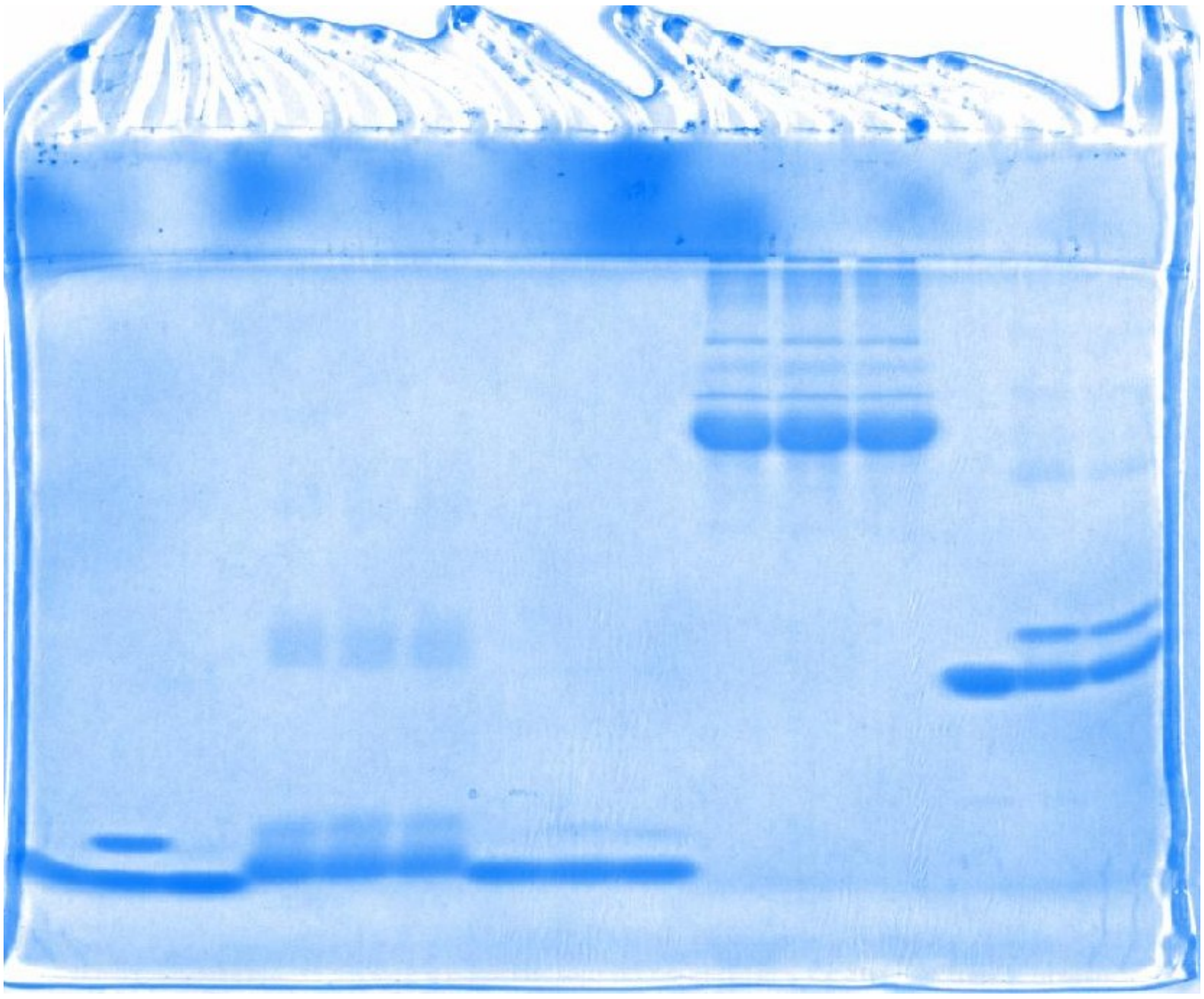

The lanes corresponding to Fig. 2B are SA only; SA + Core-AFS; SA + Rev-AFS; NeutrAvidin only; NeutrAvidin + Core-AFS; NeutrAvidin + Rev-AFS; lysozyme only; lysozyme + Core-AFS; and lysozyme + Rev-AFS. Additional lanes in the full gel are control reactions not shown in Fig. 2B.

**Source image corresponding to Fig. 2C inset**

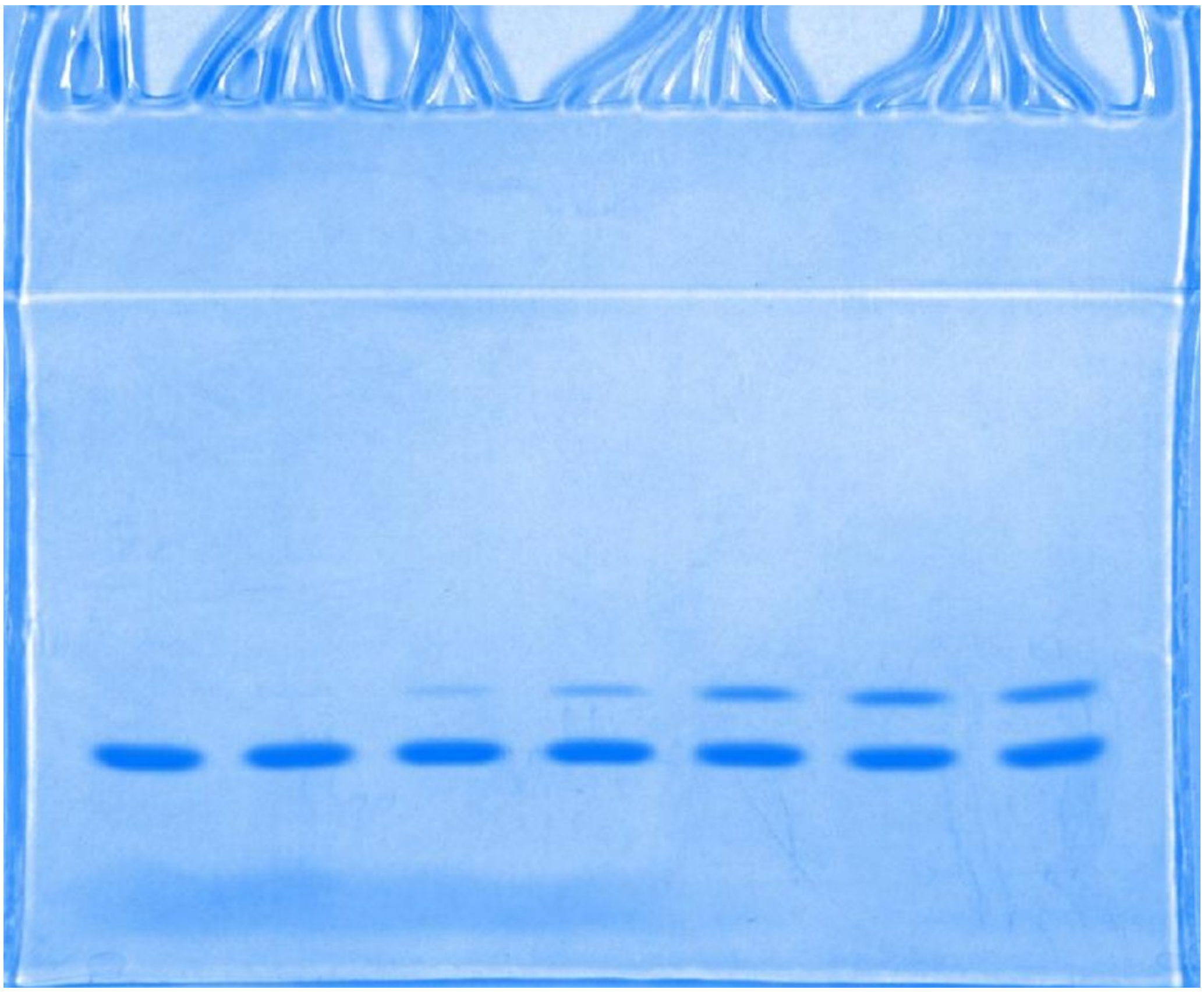

Full-gel lane order for the concentration series: SA only; SA + Core-AFS at 5, 10, 20, 40, 80, and 120 μM.

**Source image corresponding to Fig. 3A**

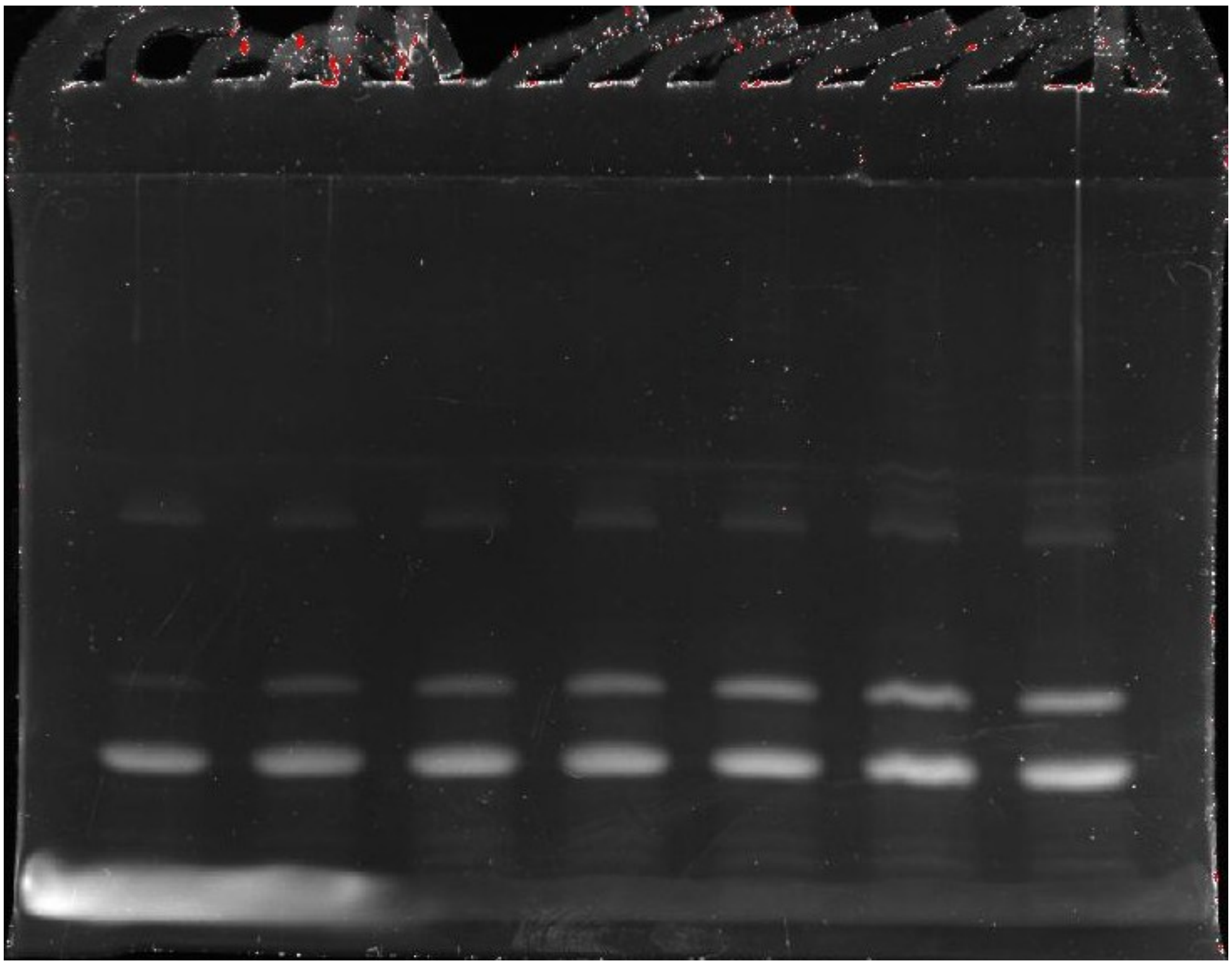

Full-gel lane order: SA + Core-AFS incubated for 1, 4, 8, 12, 24, 72, and 96 h.

## Source images corresponding to Fig. 4A (native PAGE)

SYBR Gold

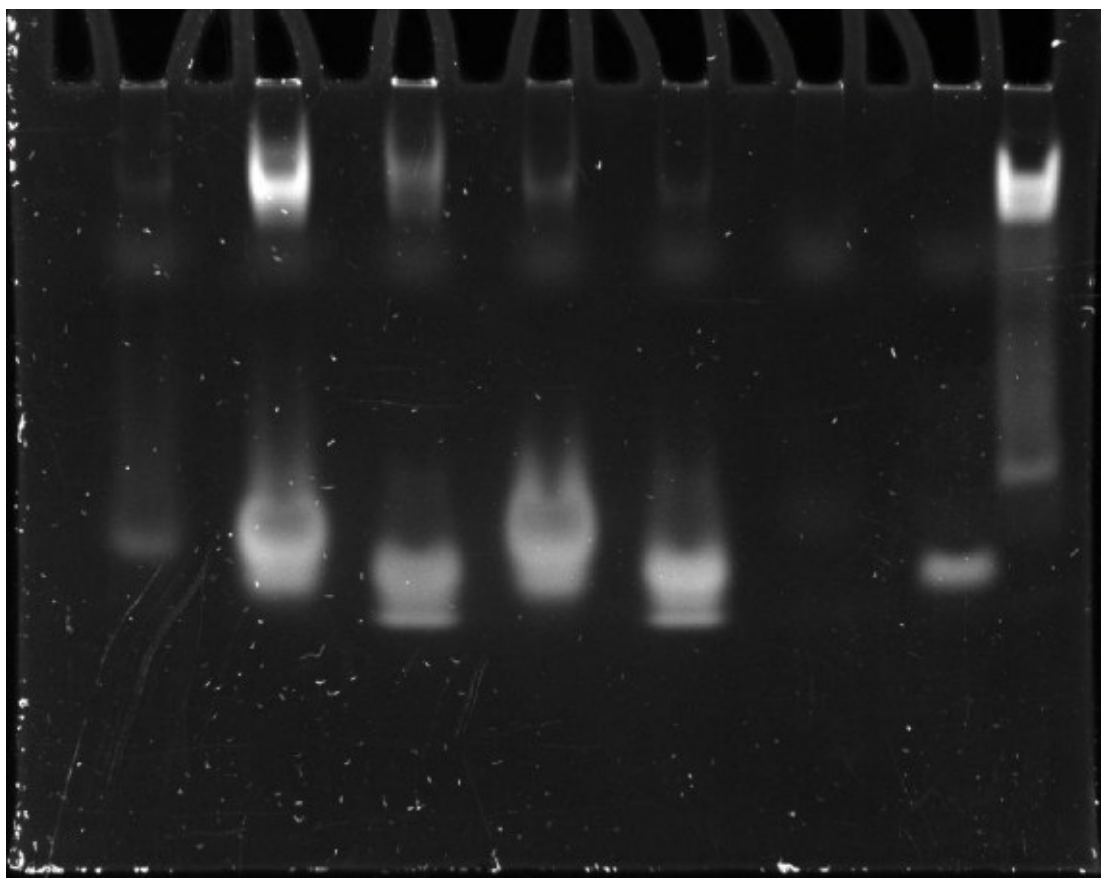

CBB

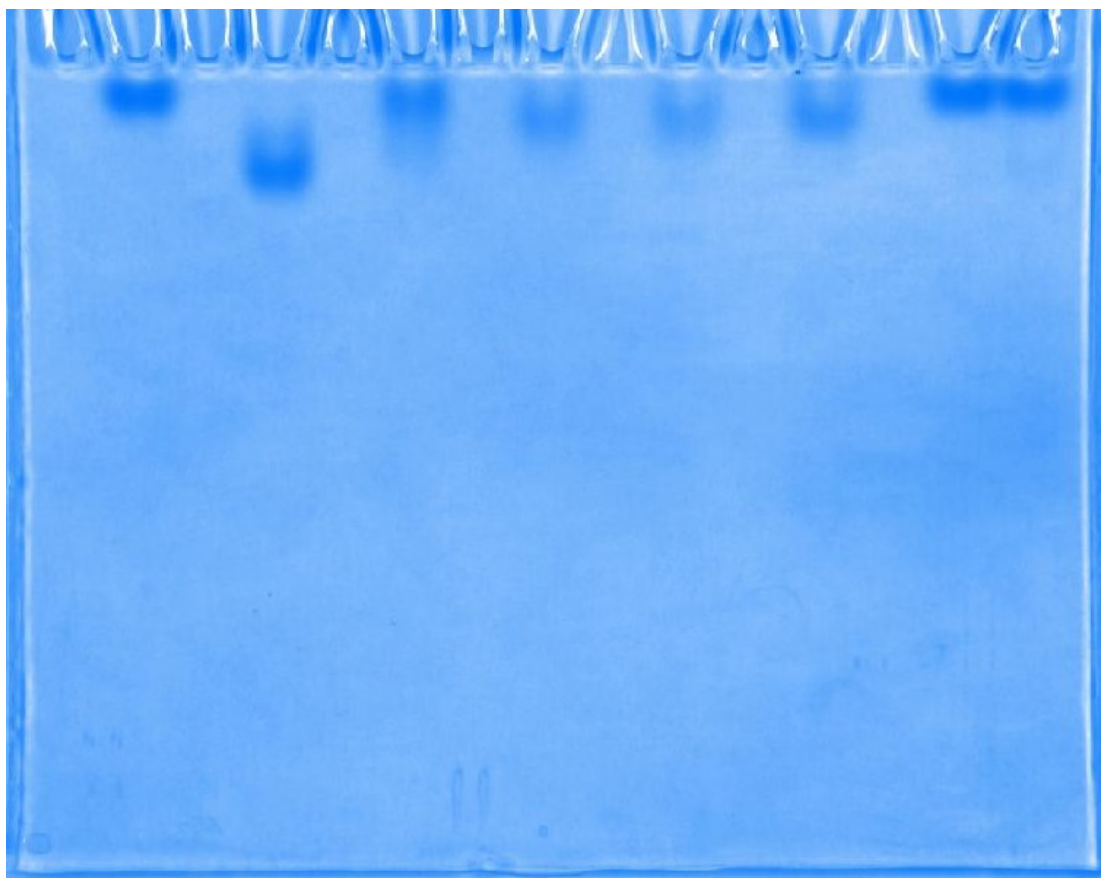

The same native gel was visualised by SYBR Gold (left) and CBB (right). Full-gel lane order: SA only; SA + Core-AFS; SA + Rev-AFS; SA pre-incubated with biotin before Core-AFS; SA pre-incubated with biotin before Rev-AFS. Additional lanes in the full gel are control reactions not shown in Fig. 4A.

**Source image corresponding to Fig. 4A (SDS-PAGE)**

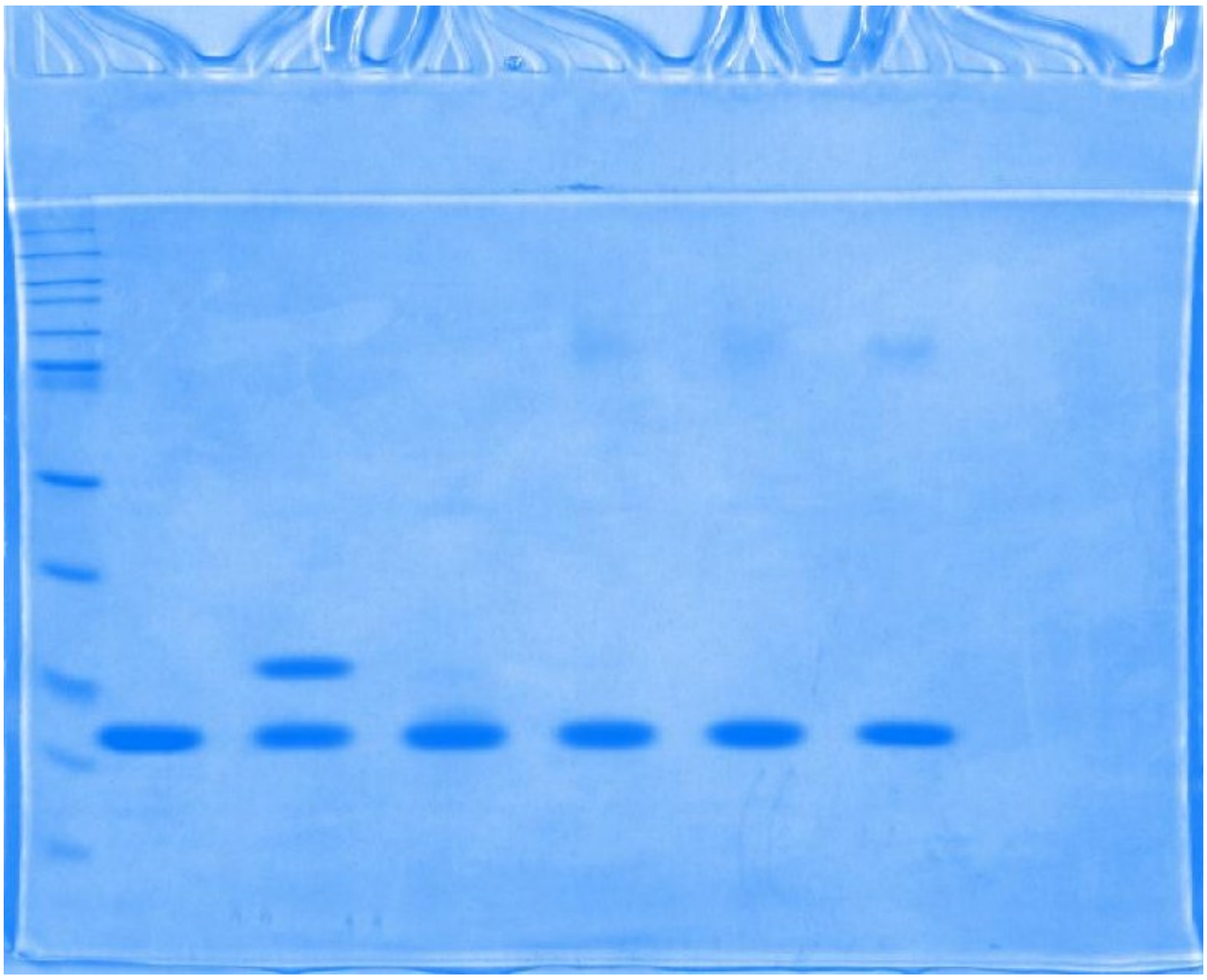

Full-gel lane order: SA only; SA + Core-AFS; SA + Rev-AFS; SA pre-incubated with biotin before Core-AFS; SA pre-incubated with biotin before Rev-AFS. Additional lanes in the full gel are control reactions not shown in Fig. 4A.

**Source image corresponding to Fig. 4C**

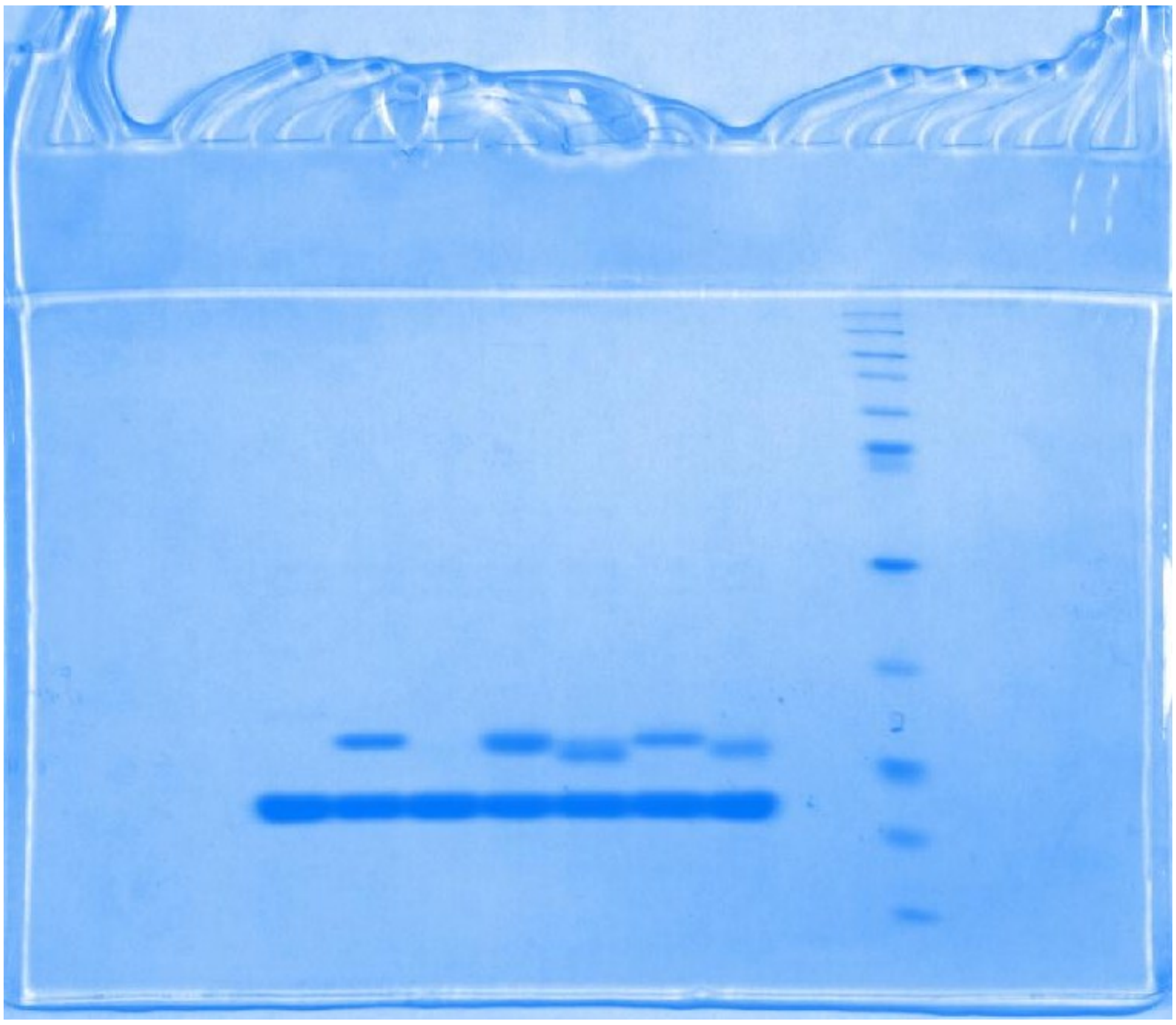

The lanes corresponding to Fig. 4C are SA only; SA + Core-AFS; SA + Rev-AFS; SA + Core-meBSF; SA + Rev-meBSF; SA + Core-BSF; and SA + Rev-BSF.

**Source image corresponding to Fig. S3**

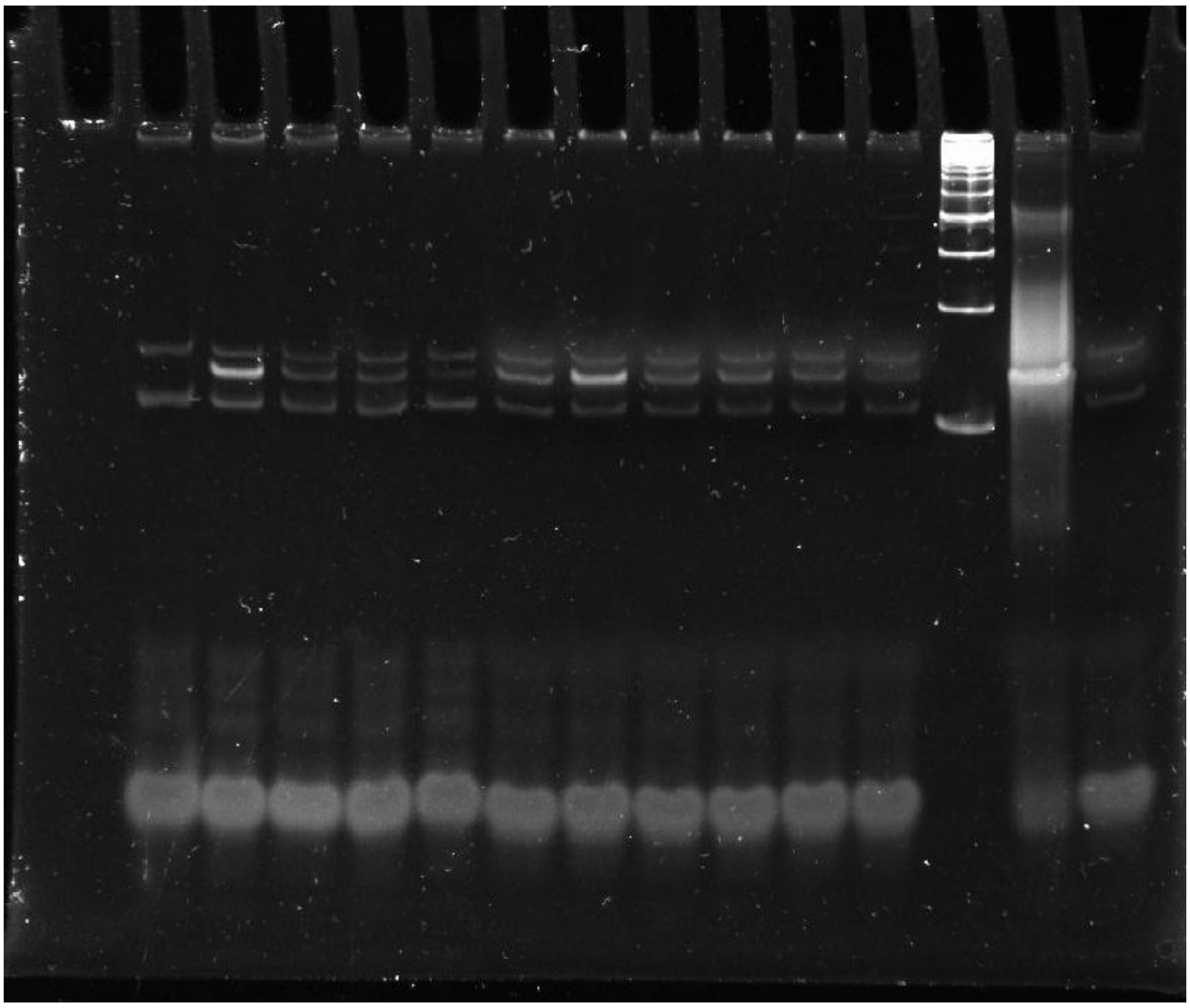

Fig. S3 was prepared from this source image; the marker and additional control lanes are retained here.

**Source image corresponding to Fig. S4**

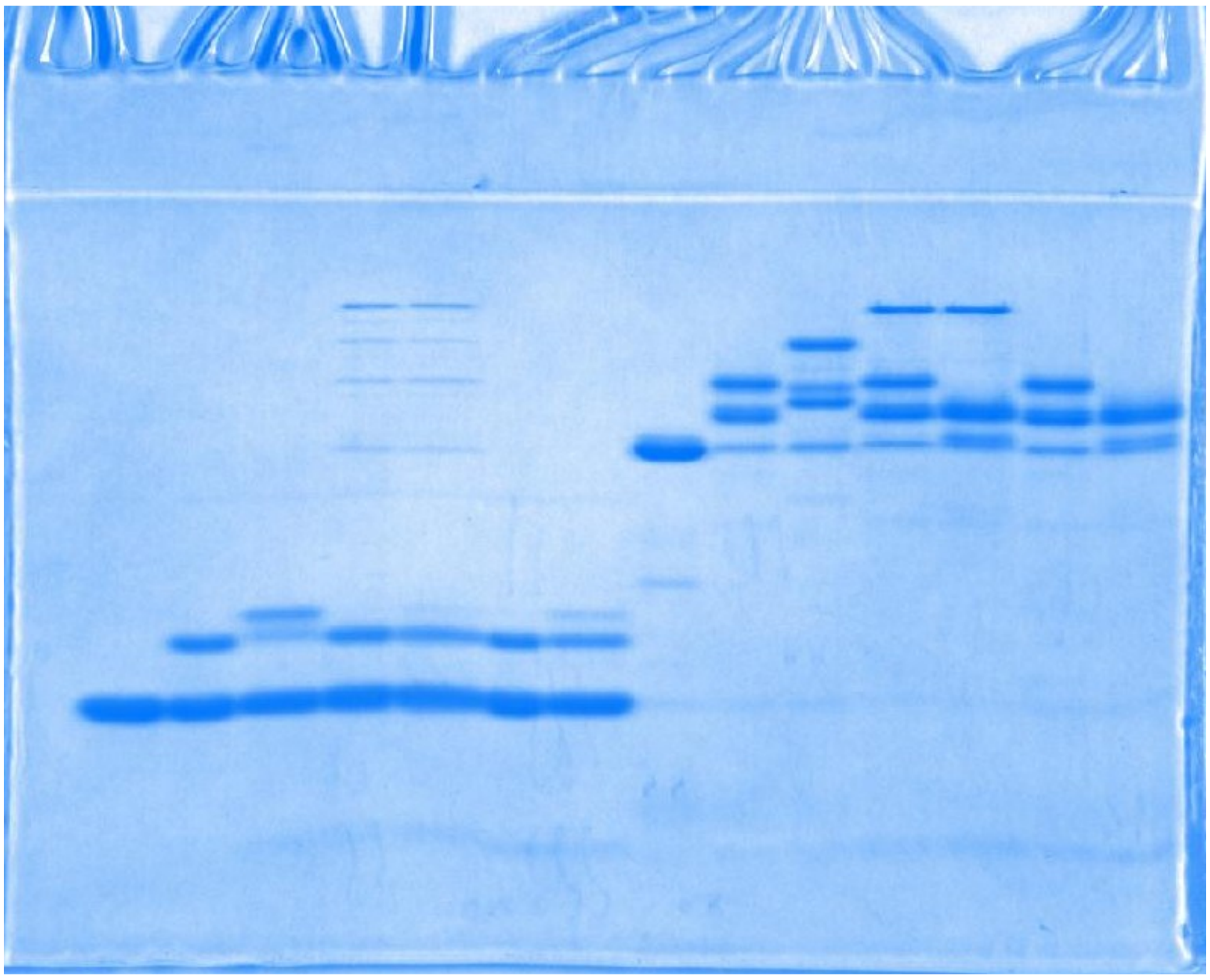

Common source image for Fig. S4. Left series: SA only; SA + Core-AFS; SA + Core-AFS + CS; SA + Core-AFS + DNase I; SA + Core-AFS + CS + DNase I; SA + Core-AFS + Benzonase; and SA + Core-AFS + CS + Benzonase. Right series: thrombin only; thrombin + $TBA_3$-BSF; thrombin + $TBA_3$-BSF + CS; thrombin + $TBA_3$-BSF + DNase I; thrombin + $TBA_3$-BSF + CS + DNase I; thrombin + $TBA_3$-BSF + Benzonase; and thrombin + $TBA_3$-BSF + CS + Benzonase.

**Source image corresponding to Fig. S6**

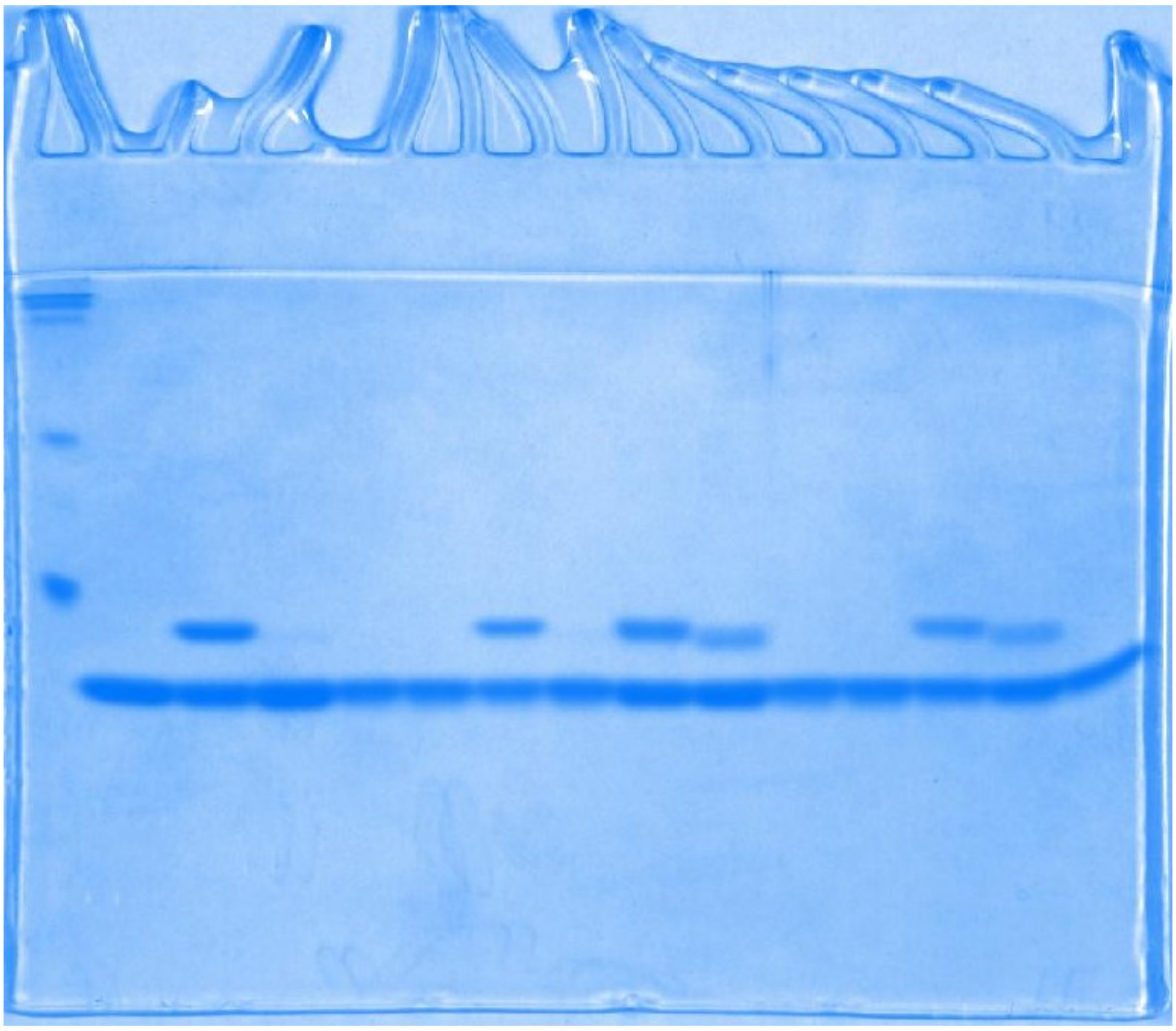

Full-gel lane order: SA only; SA + Core-AFS; SA + Rev-AFS; SA pre-incubated with biotin before Core-AFS; SA pre-incubated with biotin before Rev-AFS; SA + Core-AFS followed by biotin; and SA + Rev-AFS followed by biotin. Additional lanes in the full gel are control reactions not shown in Fig. S6.
